# Pathophysiological and therapeutic implications of C-type natriuretic peptide/cyclic GMP signaling in pulmonary fibrosis

**DOI:** 10.1172/jci.insight.196812

**Published:** 2026-01-06

**Authors:** Rene Weyer, Katharina Völker, Tamara Potapenko, Lisa Krebes, Marco Abeßer, Anna-Lena Friedrich, Eva Lessmann, Ali Khadim, Clemens Ruppert, Elie El Agha, Dalia Sheta, Andreas Beilhack, Daniel V. Santi, Eric L. Schneider, Michaela Kuhn, Swati Dabral

**Affiliations:** 1 Institute of Physiology, Julius-Maximilians University of Würzburg, Würzburg, Germany.; 2 Excellence Cluster Cardio-Pulmonary Institute (CPI), Universities of Giessen and Marburg Lung Center (UGMLC), Justus-Liebig-University Giessen, German Center for Lung Research (DZL), Giessen, Germany.; 3 Department of Internal Medicine II, Interdisciplinary Center for Clinical Research, University Hospital of Würzburg, Würzburg, Germany.; 4 ProLynx, Inc., San Francisco, California, USA.

**Keywords:** Endocrinology, Inflammation, Pulmonology, Cyclic nucleotides, Fibrosis, Guanylate cyclase

## Abstract

Activation of lung fibroblasts in response to epithelial injury and inflammation provokes pulmonary fibrosis (PF). *Endogenous* molecular brakes counteracting fibroblast activity can be targets for therapies. Preclinical studies of synthetic C-type natriuretic peptide (CNP) indicated that this hormone might provide such a brake. As shown here, CNP exerts antifibrotic effects in cultured lung fibroblasts as well as in precision cut lung slices from patients with PF, supporting clinical relevance. Therefore, augmenting or supplementing endogenous CNP could improve the treatment of such patients. To unravel whether paracrine CNP counteracts inflammation-driven PF, we studied mice with fibroblast-restricted KO of guanylyl-cyclase-B (GC-B), its cGMP-synthesizing receptor. Fibroblast GC-B-KO mice had enhanced bleomycin-induced lung inflammation, with increased expression of proinflammatory, profibrotic cytokines. Nevertheless, subsequent PF was not exacerbated. Molecular studies revealed that inflammation led to inhibition of CNP signaling in resident myofibroblasts, namely GC-B downregulation and induction of CNP/cGMP-degrading pathways. Despite this, a single s.c. injection of the recently developed long-acting CNP analog, MS~[Gln^6,14^]CNP-38, abrogated experimental lung inflammation and fibrosis. We conclude that CNP signaling in lung fibroblasts has antiinflammatory and antifibrotic effects. Attenuation of this endogenous brake participates in the pathogenesis of PF, and rescuing this pathway with long-acting CNP-analogs may have therapeutic potential.

## Abstract 2

We used a novel genetic mouse model and a recently developed stabilized long acting CNP analog, to dissect the pathophysiological and therapeutic implications of CNP/cyclic GMP signaling in inflammation-driven pulmonary fibrosis<strong>. </strong>

## Graphical abstract

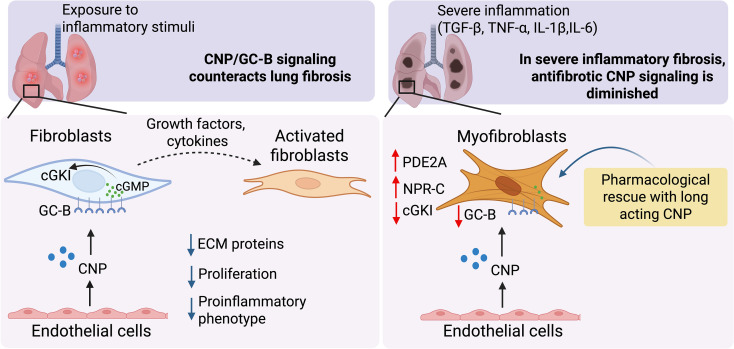

## Introduction

Pulmonary fibrosis (PF) is a progressive and often lethal disease characterized by excessive extracellular matrix (ECM) deposition, destruction of the lung architecture, and respiratory insufficiency. The pathogenesis involves recurrent or prolonged epithelial injury and an inflammatory response that triggers fibroblast transition to myofibroblasts (1). These cells build up fibrotic foci, which reduce lung compliance and obliterate the alveolar space. PF can be idiopathic (IPF), a genetically predisposed disease with a median survival time of 3 years (1). In other patients, acute or chronic inflammation of different etiologies drives progressive lung scarring. For instance, in hypersensitivity pneumonitis (HP), repeated inhalation of sensitizing antigens (e.g., organic dust, microbial particles, or animal proteins) trigger exacerbated immune responses causing epithelial injury and reactive fibrosis (2). Similarly, in interstitial lung diseases (ILDs) associated with connective tissue diseases such as systemic sclerosis or rheumatoid arthritis (CTD-ILDs), autoimmune responses and vascular injury cause epithelial damage and drive progressive PF (2). In all these entities, cytokines and growth factors such as IL-1, IL-6, TNF-α, and TGF-β promote the fibrotic process. PF is currently treated with the tyrosine kinase inhibitors pirfenidone and nintedanib, which delay disease progression but fail to target underlying pathophysiology (1). Moreover, they have many unwarranted systemic effects (1). There is a tremendous need for a better understanding of the molecular pathways driving PF as a basis for finding tractable targets.

Physiologically, fibroblasts are crucial for healing processes. They secrete ECM proteins that provide a tissue scaffold for tissue regeneration. Dissolution of this scaffold and fibroblast apoptosis restore normal tissue architecture (3). However, in the aforementioned disease conditions, cytokines and profibrotic factors maintain a constant myofibroblast phenotype, with α-smooth muscle actin (α-SMA) containing stress fibers, hyperproliferation, migration, and excessive ECM synthesis (3). Moreover, myofibroblasts secrete multiple proinflammatory factors, including TNF-α, IL-6, IL-1, Granulocyte-macrophage colony stimulating factor (GM-CSF) and C-X-C motif chemokine ligands (CXCLs) (1, 3, 4). Preventing fibroblast-myofibroblast transition obviously can improve the treatment of PF. However, while many pathogenic factors promoting this conversion have been characterized, little is known about counterregulatory pathways. Deciphering endogenous antifibrotic “brakes” might provide targets for therapies.

The hormone C-type natriuretic peptide (CNP) via its cyclic GMP-producing guanylyl cyclase-B (GC-B) receptor, has diverse auto/paracrine functions (5). These are most critical in the bone, where CNP, locally released from chondrocytes, stimulates endochondral ossification. Within the cardiovascular system, CNP is released by endothelial cells, having beneficial heart and vascular effects (5). Moreover, chronic CNP infusions or genetic CNP overexpression exerted protective actions in preclinical models of lung, heart, and liver fibrosis (5). Specifically, exogenous CNP inhibited inflammation and fibrosis in monocrotaline-induced pulmonary hypertension, LPS-induced acute lung injury, and bleomycin-induced PF (6, 7). The short half-life of CNP and consequently derived need of constant or repeated CNP infusions or injections hamper the translation of such preclinical observations into clinical trials.

Our own recent research indicated that endogenous CNP exerts paracrine antifibrotic effects, at least in the heart. Hence, mice with fibroblast-restricted GC-B deletion showed enhanced myocardial fibrosis and stiffness in response to Angiotensin II infusions or pressure overload (8). Augmentation of such antifibrotic CNP actions participates in the beneficial effects of Entresto in patients with heart failure. This drug inhibits the neprilysin-mediated degradation of the natriuretic peptides and thereby augments their endogenous levels and actions (9). Whether pharmacological reinforcement of endogenous CNP/GC-B signaling could also protect the lung and benefit patients with PF is unknown.

To unravel the possible role of paracrine CNP in the counter-regulation of pulmonary fibrotic responses, we studied mice with fibroblast-restricted GC-B deletion in the bleomycin model of PF. Fibroblast-specific ablation of CNP/GC-B signaling led to enhanced lung inflammation at early time points after bleomycin exposure. However, intriguingly, these early changes did not result in excess subsequent fibrosis. Molecular studies showed that inflammation was associated with a “loss-of-CNP-function” in resident myofibroblasts. Target tractability was indicated by the observation that a single s.c. injection of the recently developed long-acting microsphere-conjugated (MS-conjugated) stabilized CNP analog MS~[Gln^6,14^]CNP-38 (10) almost fully abrogated bleomycin-driven lung inflammation and fibrosis. Moreover, CNP and/or [Gln^6,14^]CNP-38 inhibited and even reverted the profibrotic activation of cultured human lung fibroblasts (in vitro and in situ, in precision cut lung slices), supporting the clinical relevance.

## Results

### Among various types of lung cells, GC-B expression is greatest in fibroblasts.

The CNP receptor, GC-B, is expressed in several types of cells involved in the development of PF, including vascular and immune cells (5). Supporting our focus on fibroblasts, scRNA-seq data of the Human Lung Cell Atlas (HLCA; https://cellxgene.cziscience.com/collections/6f6d381a-7701-4781-935c-db10d30de293) (11) reveal that GC-B (gene name: *NPR2*) is mainly expressed in stromal cells and at much lower levels in endothelial, epithelial and immune cells (Supplemental Figure 1, A–C; supplemental material available online with this article; https://doi.org/10.1172/jci.insight.196812DS1). Subclustering the stromal compartment into distinct fibroblast subtypes demonstrated highest *NPR2* expression in subpleural fibroblasts, followed by peribronchial and adventitial fibroblasts (Supplemental Figure 1, D and E). To validate this expression pattern at the protein level, we separated different cell populations from murine lungs using antibodies against PDGFR-α, NG2, and CD144 and magnetic-assisted cell sorting. Immunoblot analysis confirmed the separation and enrichment of PDGFR-α^+^ fibroblasts, PDGFR-β^+^ smooth muscle cells/pericytes, and CD31^+^ endothelial cells (Supplemental Figure 1F). In line with the human scRNA-seq data, GC-B protein expression was high in lung fibroblasts and low in endothelial cells.

### CNP stimulates antifibrotic GC-B/cGMP signaling in cultured lung fibroblasts from patients with IPF.

**Figure 1:**
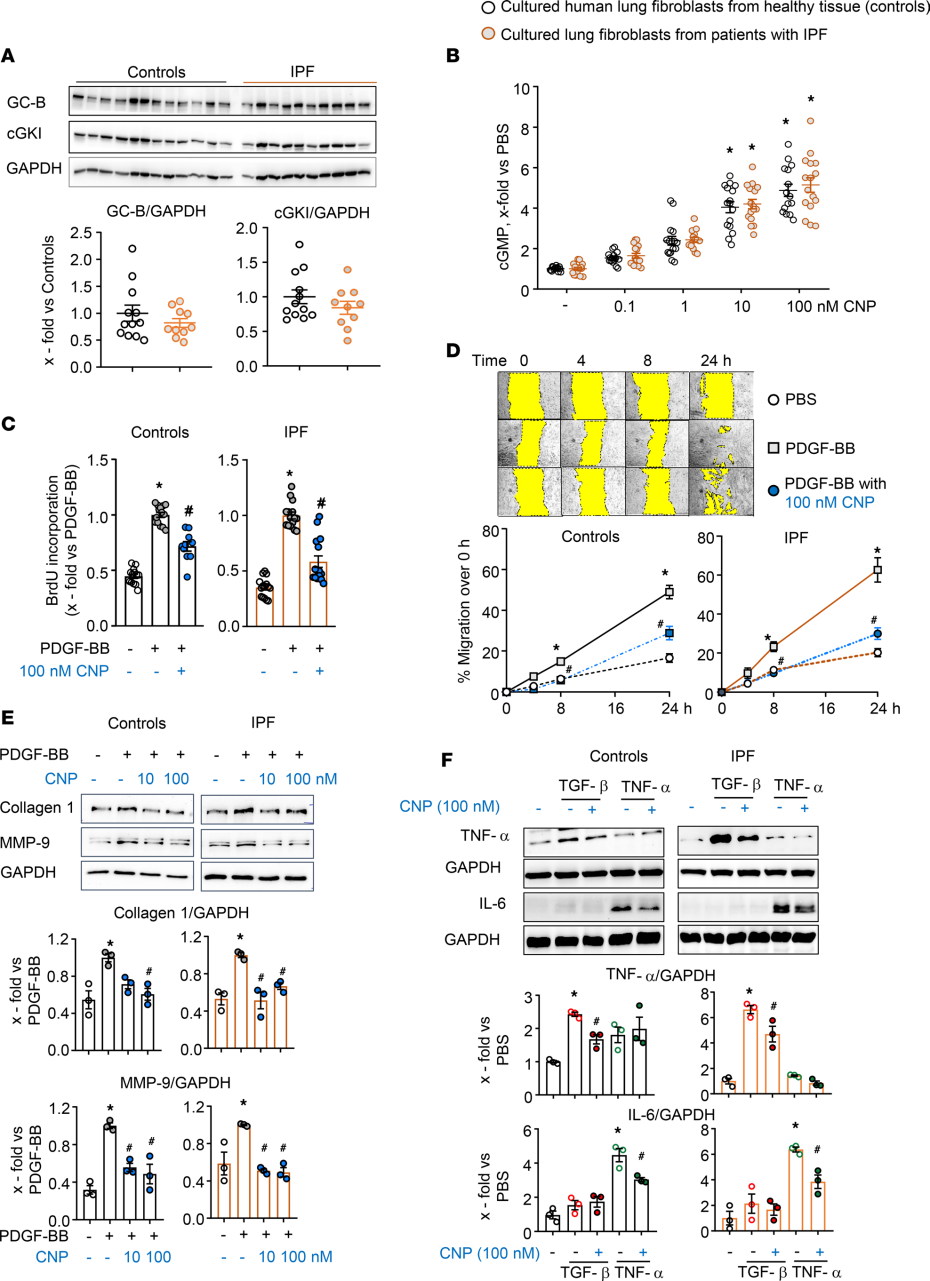
CNP, via GC-B/cGMP signaling, attenuates the profibrotic and proinflammatory activation of cultured human lung fibroblasts, and such effects are preserved in IPF fibroblasts. (**A**) GC-B and cGKI expression in control and IPF fibroblasts (immunoblots). (**B**) Effects of CNP (0.1–100 nM, 15 minutes) on intracellular cGMP contents (detected by radioimmunoassay) of control and IPF lung fibroblasts in presence of the PDE inhibitor 3-Isobutyl-1-methylxanthin *(*0.5 mM IBMX). (**C**–**E**) Effects of CNP pretreatment (30 minutes) on PDGF-BB–induced (50 ng/mL, 24 hours) proliferation (BrdU incorporation; **C**), migration (scratch assay; **D**), and expression levels of collagen 1 and MMP-9 (immunoblots; **E**). (**F**) Effects of CNP pretreatment (30 minutes) on TGF-β–induced (2 ng/mL, 24 hours) and TNF-α–induced (10 ng/mL, 24 hours) expression of TNF-α and IL-6, respectively (immunoblots). The sample number for each experiment (*n*) varied between 3 and 16 and is indicated by the number of data points in each histogram. The scratch assays were performed with 12–16 wells from 3 biological replicates from each group. Significance was determined by unpaired Student’s *t* test (**A**), 1-way ANOVA (**B**, **C**, **E**, and **F**), and 2-way ANOVA (**D**). *P < 0.05 versus PBS (–), ^#^*P* < 0.05 versus PDGF-BB, TGF-β, or TNF-α.

To assess the functional and possible clinical relevance of the GC-B expression data, we studied CNP/GC-B/cGMP signaling in cultured human lung fibroblasts. Healthy “control” lung fibroblasts were derived from unused explanted donor lungs or from histologically unaltered lung regions surrounding resected tumors. “IPF fibroblasts” were derived from end-stage patients undergoing lung transplantation. In comparison with control fibroblasts, IPF fibroblasts had unaltered baseline proliferation rates, as shown by bromodeoxyuridine (BrdU) incorporation (Supplemental Figure 2A). However, in agreement with published studies (12), the proliferative effect of platelet-derived growth factor BB (PDGF-BB; 50 ng/mL, 24 hours) was enhanced (Supplemental Figure 2A). To study migration, fibroblasts were seeded in 24-well plates and grown until confluency. A scratch was made followed by PDGF-BB (50 ng/mL) or PBS (vehicle) treatment and wound closure was monitored for 24 hours. As shown, IPF fibroblasts showed accelerated baseline and PDGF-BB–stimulated migration (Supplemental Figure 2B). Hence, cultured IPF fibroblasts “intrinsically” exhibit increased migration and proliferation (present studies) as well as differentiation into a contractile phenotype (12), with enhanced responses to growth factors such as TGF-β and PDGF-BB. Notably, the expression levels of the GC-B receptor and of its downstream target cGMP-dependent Protein Kinase I (cGKI) were similar in control and IPF fibroblasts (immunoblots in Figure 1A). Accordingly, in both groups, CNP raised intracellular cGMP levels in a similar concentration-dependent manner (Figure 1B).

To evaluate whether CNP moderates the profibrotic effects of growth factors and cytokines, fibroblasts were pretreated with CNP (100 nM, 30 minutes) prior to stimulation for 24 hours. CNP significantly reduced the proliferative and promigratory actions of PDGF-BB (50 ng/mL), and these inhibitory CNP effects were preserved and even slightly enhanced in IPF fibroblasts (Figure 1, C and D). The PDGF-BB–stimulated expression of the ECM proteins collagen 1 and matrix metalloproteinase-9 (MMP-9) was also attenuated by 10 and 100 nM CNP in both control and IPF cells (immunoblots in Figure 1E). Moreover, in both groups, CNP (100 nM, 30 minutes pretreatment) attenuated the TGF-β–induced expression (2 ng/mL, 24 hours) of the myofibroblast marker, collagen triple helix repeat containing protein 1 (Cthrc1) (Supplemental Figure 3). Lastly, we examined the effect of CNP on the expression of the proinflammatory “fibrokines” TNF-α and IL-6. As shown in Figure 1F, TGF-β (2 ng/mL, 24 hours) and TNF-α (10 ng/mL, 24 hours) augmented the expression of TNF-α and IL-6, respectively, in control and even more in IPF fibroblasts. CNP (100 nM, 30 minutes pretreatment) reduced such proinflammatory activation in both groups. We conclude that the CNP/GC-B/cGMP pathway exerts antifibrotic and antiinflammatory effects in cultured human lung fibroblasts. Such effects are preserved in fibroblasts from patients with IPF in vitro.

### Development and characterization of a fibroblast-specific GC-B–KO mouse line.

**Figure 2:**
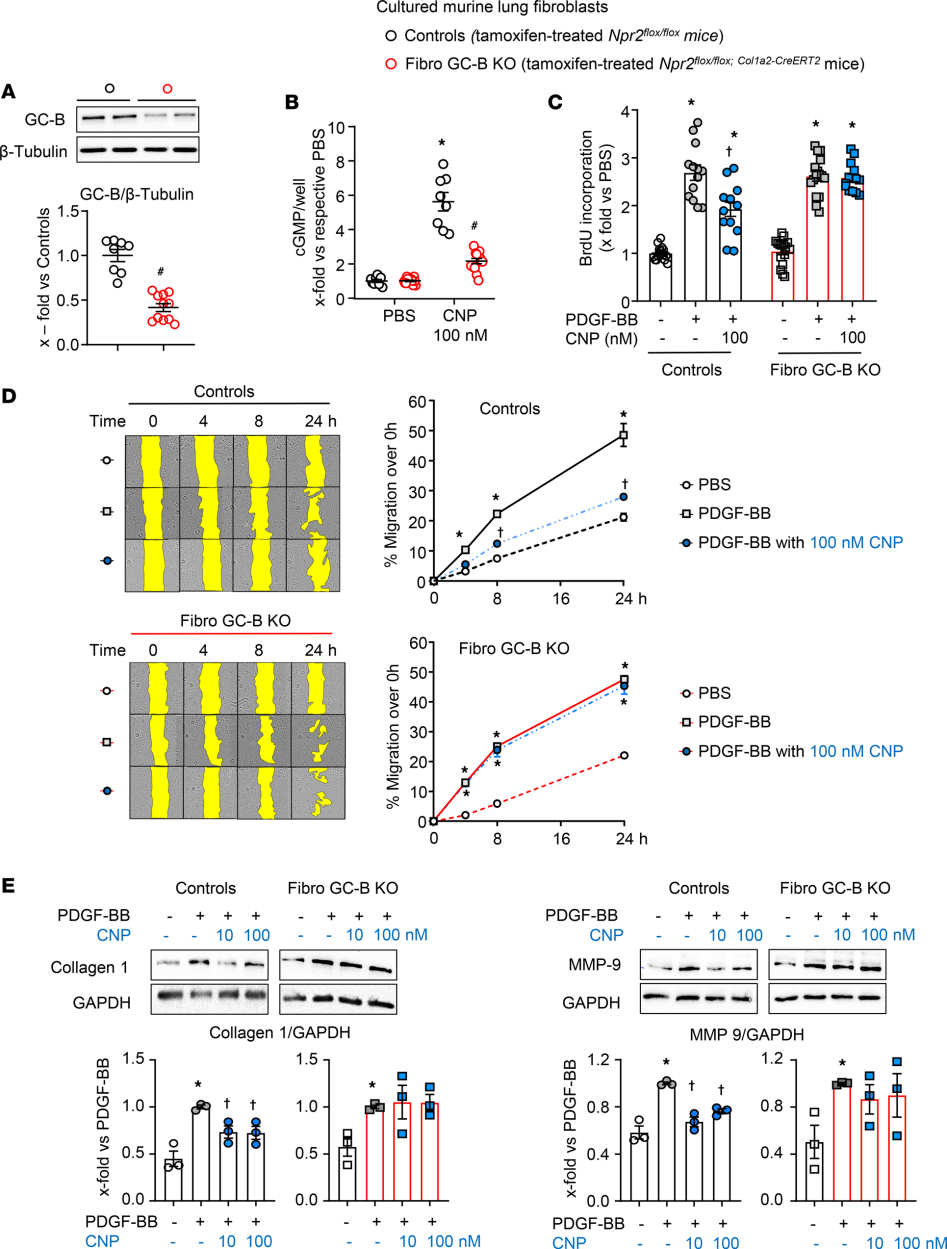
Comparison of GC-B expression and signaling in lung fibroblasts isolated from tamoxifen-treated *Npr2^fl/fl^* (controls) and *Npr2^fl/fl^;Col1a2-Cre^ERT2^* littermate mice (Fibro GC-B KO). Cultured fibroblasts were studied at passages 2 and 3. (**A**) Immunoblot: Expression of GC-B was normalized to β-tubulin and calculated as x-fold from the average of controls. (**B**) Effects of CNP on intracellular cGMP contents were determined by radioimmunoassay and calculated as x-fold versus PBS. (**C**–**E**) Effect of CNP pretreatment (30 minutes) on PDGF-BB–induced (50 ng/mL, 24 hours) proliferation (**C**), migration (**D**), and collagen 1 as well as MMP-9 expression (**E**) of such control and GC-B–deficient murine lung fibroblasts. The sample number for each experiment (*n*) varied between 3 and 14 and is indicated by the number of data points in each histogram. The scratch assays were performed with 8–14 wells from 3 biological replicates from each group. Significance was determined by unpaired 2-tailed Student’s *t* test (**A**), 2-way ANOVA (**B**–**D**), and 1-way ANOVA (**E**). *P < 0.05 versus PBS ([–], in all panels), ^#^*P* < 0.05 versus control mice (**A** and **B**); and ^Ɨ^*P* < 0.05 versus PDGF-BB (**C**–**E**).

To unravel whether endogenous paracrine CNP/GC-B signaling counterregulates the pathological activation of lung fibroblasts, we utilized a recently reported transgenic mouse line with tamoxifen-induced fibroblast-restricted GC-B deletion (Fibro GC-B KO: *Npr2^fl/fl;Col1a2-CreERT2^* mice) (8). To study the efficiency of the genetic GC-B deletion in lung fibroblasts, such cells were isolated from tamoxifen-treated Fibro GC-B KO and Cre-negative littermate mice (controls: *Npr2^fl/fl^*). Cultured lung fibroblasts were passaged twice and then plated for experiments. Western blotting demonstrated an approximately 50% reduction of GC-B expression in GC-B–KO lung fibroblasts (Figure 2A). CNP (100 nM) increased cGMP contents of control fibroblasts by approximately 6-fold and by only approximately 2-fold in KO fibroblasts (Figure 2B). To investigate how inhibited CNP/GC-B/cGMP signaling affects the interactions with PDGF-BB, we performed functional assays. PDGF-BB (50 ng/mL, 24 hours) enhanced the proliferation, migration, and collagen 1 as well as MMP-9 expression levels of murine lung fibroblasts without genotype-dependent differences (Figure 2, C–E). As shown, CNP significantly attenuated these effects of PDGF-BB in control but not in GC-B–KO fibroblasts.

As recently published (8), in contrast to mice with global GC-B deletion (5), such Fibro GC-B–KO mice have normal Mendelian inheritance, life span, and skeletal growth. Under baseline and sham conditions, arterial blood pressure, cardiac and lung function, and cardiac and lung interstitial collagen fractions were not different between control and KO littermates from both sexes.

### Mice with fibroblast-restricted GC-B inhibition show enhanced early lung inflammation in response to bleomycin.

To explore the effects of a fibroblast-specific inhibition of CNP signaling on pathological lung inflammation and subsequent fibrosis, control and Fibro GC-B–KO mice received bleomycin (0.75 IU/kg bodyweight [BW]) by intratracheal administration under anesthesia. Bleomycin injures the airway epithelium and, thereby, provokes a rapid inflammatory response, followed by subsequent reactive interstitial fibrosis (7, 13). Thereby, this model resembles inflammation-driven fibrosis in patients.

**Figure 3:**
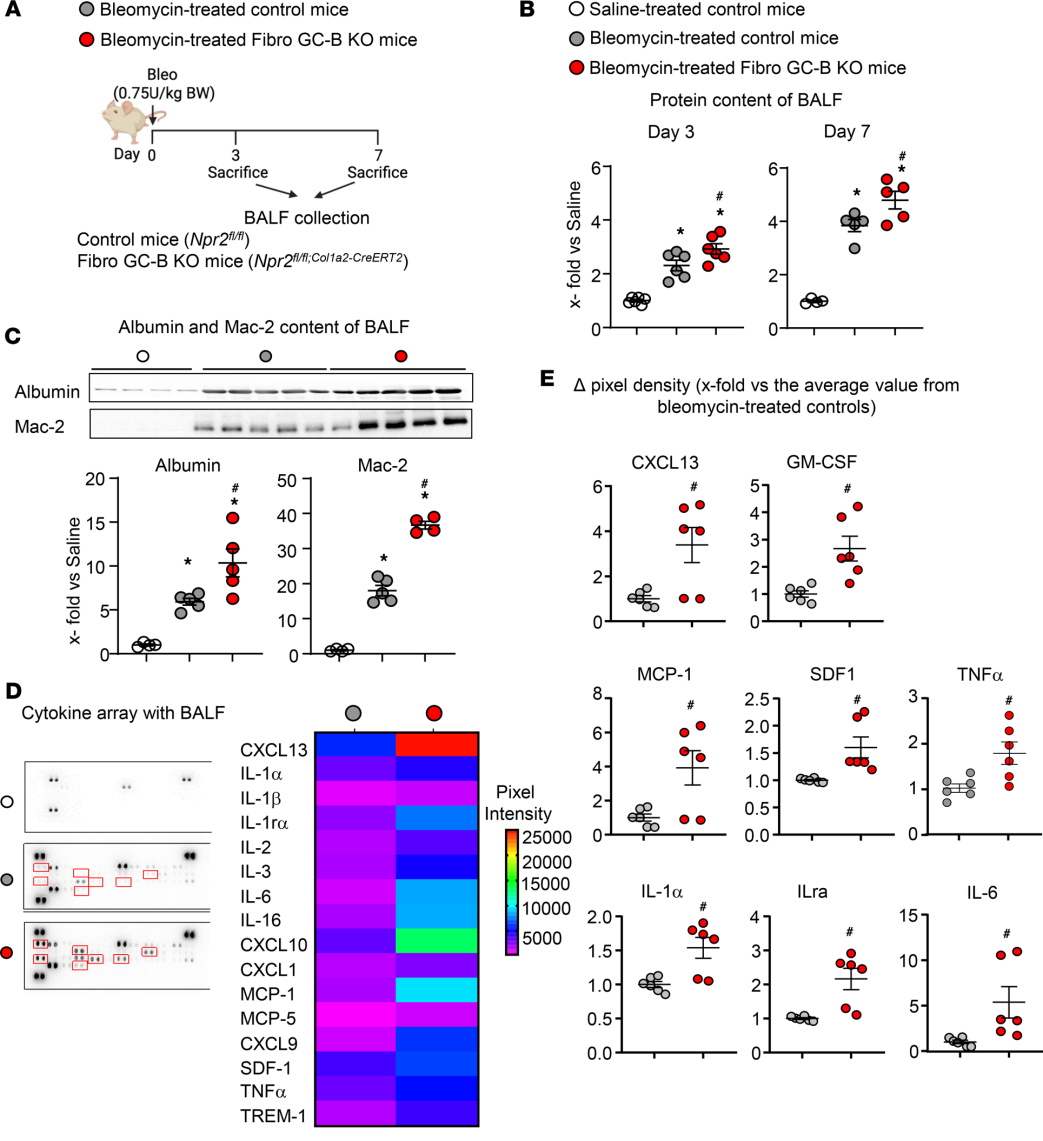
Fibroblast-restricted GC-B deletion exacerbates bleomycin-induced lung inflammation in mice. (**A**) Schematic illustration of these studies performed in control and Fibro GC-B KO littermate mice 3 and 7 days after bleomycin administration. (**B**) Plasma leakage was evaluated 3 and 7 days after bleomycin treatment by determination of the protein content in bronchoalveolar lavage fluid (BALF) samples (by BCA assay). (**C**) Albumin and Mac-2 levels were evaluated in equal volumes of BALF obtained 7 days after bleomycin treatment (immunoblot). (**D** and **E**) Cytokine contents of BALF samples obtained 7 days after bleomycin treatment were evaluated with a commercially available mouse cytokine array. **D** shows representative cytokine array membranes probed with BALF from saline- or bleomycin-treated control mice as well as from bleomycin-treated Fibro GC-B–KO littermate mice. The heatmap depicts cytokines that were detected at markedly different levels in BALF from the 2 genotypes (mean-normalized values are shown, with red color indicating high, and blue indicating low values). (**E**) Quantitative evaluation of pixel densities of representative cytokines differing between bleomycin-treated control and KO mice. In **B** and **C**, each data point represents an individual study mouse; **E** shows replicates from 3 mice per group. Significance was tested by 1-way ANOVA (**B** and **C**) and Student’s unpaired *t* test (**E**). *P < 0.05 versus vehicle; ^#^*P* < 0.05 versus control mice.

In the first series of experiments, mice were euthanized 3 and 7 days after bleomycin administration (scheme in Figure 3A), to characterize the inflammatory phase through analyses of bronchoalveolar lavage fluid (BALF) (13), as well as the molecular signatures of resident fibroblasts isolated from the lung parenchyma. BALF samples were used to quantify extravasated proteins (i.e., albumin) with a regular BCA assay (Figure 3, B and C) and by immunoblotting (Figure 3C). As shown in Figure 3, B and C, the bleomycin-treated mice exhibited greater protein, especially albumin, levels in BALF than saline-treated mice. In control mice, such “plasma leakage” was increased by 3-fold on day 3 and by 4- to 5-fold on day 7 after bleomycin instillation (as compared with saline). These inflammatory responses were increased in Fibro GC-B–KO mice (Figure 3, B and C). The findings were corroborated by immunoblot analyses of Mac-2, a marker for macrophages (Figure 3C). Mac-2 was barely detectable in BALF from vehicle-treated mice, but the levels rose by nearly 20-fold in BALF from bleomycin-treated control mice (day 7), suggesting a strong activation and/or transepithelial migration of tissue macrophages. Notably, in BALF of bleomycin-treated Fibro GC-B–KO mice, the levels of Mac-2 in BALF rose nearly 40-fold (Figure 3C).

To further characterize the inflammatory response 7 days after bleomycin treatment, we used a cytokine array detecting 40 different cytokines (ARY006, R&D Systems). As illustrated in Figure 3D, BALF from bleomycin-treated control mice contained numerous proinflammatory cytokines and chemokines, which were all undetectable in saline-instilled mice. This response to bleomycin was much greater in the Fibro GC-B–KO mice (Figure 3D). Supplemental Figure 4 provides an overview of all changed cytokines. For a more comprehensive appreciation of the genotype-dependent changes, Figure 3E depicts quantitative evaluations of the cytokines, which are known to be secreted by activated myofibroblasts: CXCL13, GM-CSF, IL-1α, IL-6, monocyte chemoattractant protein-1 (MCP-1)/CCL2, stromal cell-derived factor 1 (SDF-1/CXCL12), IL-1 receptor antagonist (IL-1Ra) and TNF-α (4, 13–22). Notably, the levels of all these fibrokines were significantly greater in the BALF of bleomycin-treated Fibro GC-B–KO mice than in BALF from their control littermates. Collectively, these results confirm that bleomycin-induced pulmonary inflammation was exacerbated in Fibro GC-B–KO mice.

### Mice with fibroblast-restricted GC-B inhibition show unaltered lung fibrosis in response to bleomycin.

**Figure 4:**
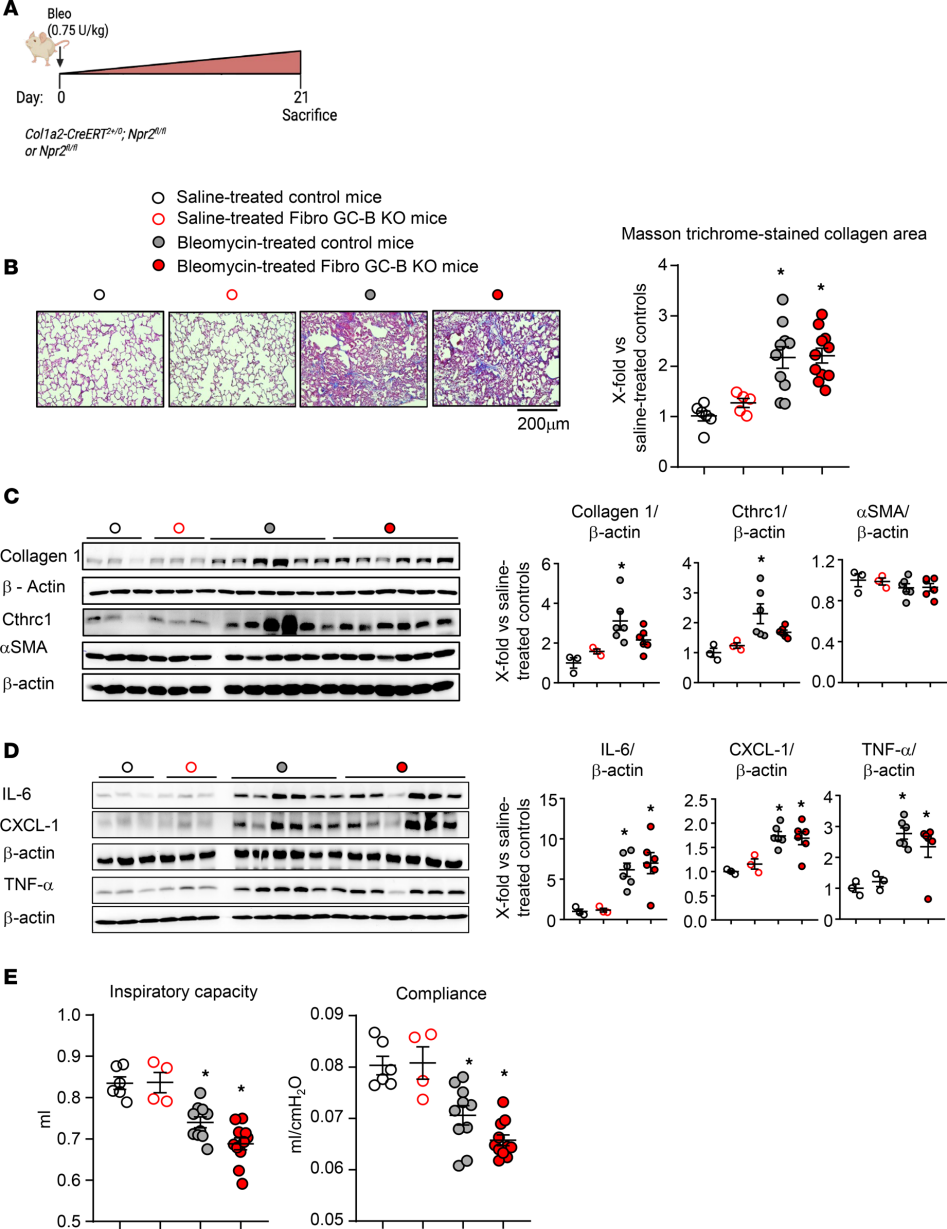
Fibroblast-restricted GC-B deletion does not significantly alter bleomycin-induced lung fibrosis and dysfunction in mice. (**A**) Schematic illustration of these studies in control and Fibro GC-B–KO littermate mice 21 days after bleomycin administration. (**B**) Collagen deposition was determined by Masson’s trichrome staining of lung paraffin sections, followed by quantification with ORBIT. The mean value from quantification of 2 sections per mouse was taken (10–15 fields per lung were evaluated). Collagen areas (in percent from corresponding total section areas) are presented as x-fold from the average value of saline-instilled control mice. (**C** and **D**) Immunoblots: pulmonary collagen 1, Cthrc1, α-SMA (**C**), IL-6, CXCL-1, and TNF-α expression levels (**D**) were normalized to β-actin and calculated as X-fold from the average value of saline-treated control mice. (**E**) Lung inspiratory capacity and compliance were evaluated in anesthetized mice with a Flexivent system. For **B** and **E**: *n* = 6 (Controls, saline), 4–5 (KO, saline), 10 (Controls, bleomycin), and 11 mice (KO, bleomycin). For **C** and **D**: *n* = 3 (Controls, saline), 3 (KO, saline), 6 (Controls, bleomycin) and 6 mice (KO, bleomycin). Significance was tested by 2-way ANOVA. *P < 0.05 versus saline.

To characterize the postinflammatory fibrotic phase, mice were studied 21 days after bleomycin administration (scheme in Figure 4A) (22). Masson’s trichrome staining of lung sections together with immunoblot studies revealed significant interstitial collagen deposition in lungs from bleomycin-treated mice. Unexpectedly, we did not observe differences between control and Fibro GC-B–KO mice (Figure 4, B and C). Together with collagen 1, pulmonary Cthrc1 expression was also upregulated in bleomycin-treated mice, without differences between genotypes. In fact, levels trended lower in the KO mice (Figure 4C). Pulmonary α-SMA expression was not different between treatment groups or genotypes (Figure 4C). We assume that the expression of α-SMA in vascular and airway smooth muscle cells masked the bleomycin-induced increase in fibroblast-specific α-SMA.

To characterize the inflammatory response, we studied the pulmonary expression levels of IL-6, CXCL-1, and TNF-α by immunoblot. As shown in Figure 4D, all 3 cytokines were similarly increased in lungs from bleomycin-treated control and KO mice.

The effect on lung function was evaluated with a Flexivent system (in anesthetized mice, immediately before necropsy). In line with the histology, no differences in lung capacity and compliance were observed between saline-treated control and Fibro GC-B–KO mice (Figure 4E), demonstrating that the GC-B knockdown did not affect baseline lung morphology and function. Bleomycin-treated mice showed a significant decline of inspiratory capacity and compliance. These functional changes were slightly more pronounced in Fibro GC-B–KO mice than in controls, but this difference did not reach statistical significance (Figure 4E).

### Bleomycin-induced lung inflammation impairs the CNP/GC-B signaling pathway in resident lung myofibroblasts.

**Figure 5:**
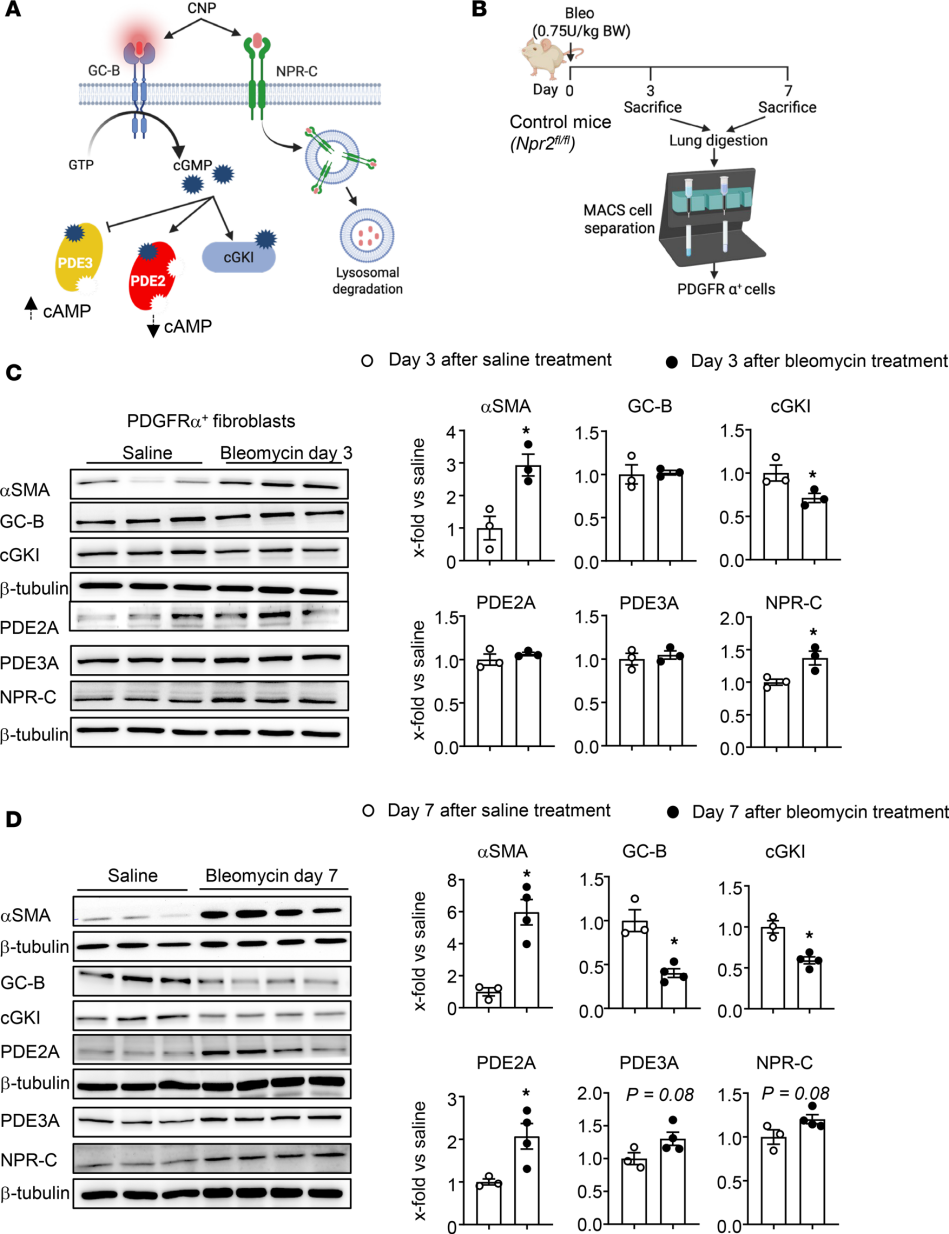
Bleomycin-induced lung inflammation impairs the CNP/GC-B signaling pathway in resident lung myofibroblasts. (**A**–**D**) Schematic illustrations of the CNP/GC-B/cGMP signaling cascade (**A**) and of the animal protocol (**B**) used for these studies in control (*Npr2^fl/fl^*) mice 3 days (**C**) and 7 days (**D**) after bleomycin or vehicle administration. Lungs were digested with dispase and the isolated pulmonary PDGFR-α^+^ fibroblasts were enriched for immunoblot studies. Due to low cell yield, eventually, lung fibroblasts from 2 mice had to be combined for target protein determinations. The expression levels of α-SMA, GC-B, cGKI, PDE2A, PDE3A, and NPR-C were normalized to β-tubulin and calculated as the average value from vehicle-treated (saline-treated) mice (*n* = 3–4 samples from 6–8 mice per group). Significance was tested by Student’s unpaired *t* test. *P < 0.05 versus vehicle.

The main results of the previous sections can be summarized as follows: (a) synthetic CNP, via GC-B/cGMP signaling, inhibits the profibrotic activation of cultured human and murine lung fibroblasts; (b) transgenic mice with inhibited CNP/GC-B signaling in fibroblasts react to bleomycin with enhanced lung inflammation; and (c) despite this, such Fibro GC-B–KO mice do not have exacerbated reactive lung fibrosis and dysfunction. In view of this discrepancy, we hypothesized that bleomycin-induced lung inflammation inhibits the CNP/GC-B signaling pathway and/or the expression and activity of cGMP-modulated or -modulating proteins in resident lung (myo)fibroblasts. CNP/GC-B, via cGMP, can activate cGKI and the dual cGMP/cAMP-degrading phosphodiesterase (PDE) 2A. In addition, cGMP inhibits the cAMP-degrading PDE 3A (5). Furthermore, apart from GC-B, CNP binds with high affinity to the Natriuretic Peptide Receptor C (NPR-C), a “clearance” receptor mediating the cellular internalization and degradation of natriuretic peptides (5). Figure 5A provides a scheme of these pathways. To evaluate the expression of these signaling proteins in resident lung (myo)fibroblasts, the lungs from control mice euthanized 3 or 7 days after bleomycin or vehicle treatment were enzymatically digested, fibroblasts were enriched with magnetic beads coated with antibodies against the fibroblast protein PDGFR-α, and they were immediately lysed with RIPA buffer for immunoblot studies (scheme in Figure 5B).

As shown in Figure 5C, in PDGFR-α^+^ fibroblasts isolated 3 days after bleomycin treatment, the expression of α-SMA was increased by approximately 3-fold (as compared with vehicle), indicating the starting differentiation to myofibroblasts. At this early point, the expression levels of GC-B, PDE2A, and PDE3A were not different between fibroblasts from vehicle- and bleomycin-treated mice. However, NPR-C expression was significantly increased, and cGKI expression levels were mildly but significantly reduced.

In activated α-SMA^+^ lung myofibroblasts obtained from mice 1 week after bleomycin instillation, such alterations of cGKI and NPR-C levels were maintained and accompanied by altered expression of the whole CNP signaling machinery (Figure 5D). PDE3A and especially PDE2A levels were upregulated. Most importantly, GC-B expression was significantly reduced by about 50% (compared with saline). In fact, the reduction of fibroblast GC-B expression observed in the bleomycin-treated control mice was like the genetic reduction achieved in the Fibro GC-B–KO mice (please compare Figure 5D and Figure 2A).

Collectively, these data reveal that, together with the inflammatory process, fibroblast-to-myofibroblast activation progresses from day 3 to day 7 after bleomycin instillation. While on day 3, the CNP signaling pathway in resident (myo)fibroblasts was mostly preserved, on day 7, the expression of molecules mediating CNP/cGMP effects (GC-B and cGKI) was diminished. Moreover, pathways that degrade CNP (NPR-C) and cGMP as well as cAMP (PDEs 2A and 3A) were induced. This indicates “loss-of-CNP-function” in activated lung myofibroblasts of bleomycin-treated control mice and may explain why bleomycin-induced lung fibrosis did not differ between control and Fibro GC-B KO littermates.

### Cytokines involved in lung inflammation and fibrosis impair the CNP/GC-B signaling pathway in cultured human lung fibroblasts.

To characterize the upstream factors altering the CNP/GC-B signaling pathway, we tested the effect of relevant cytokines (1, 13, 15, 18) on cultured normal human lung fibroblasts. Cells were treated with TGF-β, TNF-α, MCP-1, IL-1β, or IL-6 (all at 10 ng/mL; except IL-6, used at 50 IU/mL) for 48 hours and then lysed for immunoblot studies. Supplemental Figure 5 illustrates that TGF-β and TNF-α reduced the expression levels of GC-B and cGKI; all cytokines greatly increased the expression of PDE2A, and TGF-β additionally elevated the levels of PDE3A; TNF-α and IL-1β robustly induced the expression of NPR-C. Considering that, in pulmonary inflammation, the resident fibroblasts are exposed to a combination of these cytokines (13–22), this may account for the complex remodeling of the CNP signaling pathways in the bleomycin model during the inflammatory phase (Figure 5).

**Figure 6:**
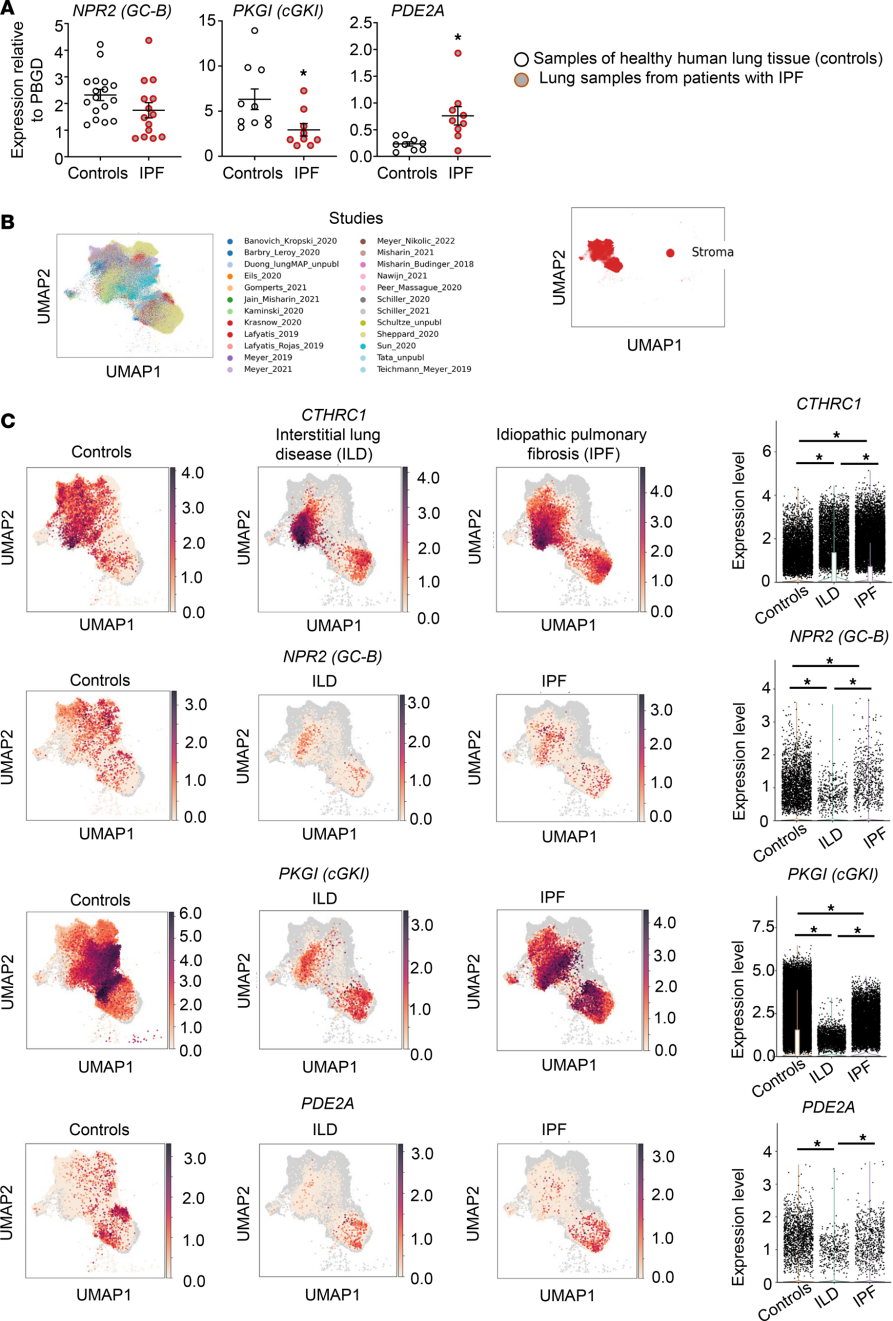
Altered mRNA expression of components of the CNP/GC-B signaling pathway in whole lung samples and lung stromal cells from patients with PF. (**A**) The mRNA expression levels of *NPR2* (GC-B), *PKGI* (cGKI), and PDE2A in samples of healthy human lung tissue and lung samples from patients with IPF were studied by qPCR. The expression was normalized to porphobilinogen deaminase (PBGD). The sample number for each experiment (*n*) varied between 10 and 16 and is indicated by the number of data points in each graph. Significance was tested by Student’s unpaired *t* test. *P < 0.05 versus controls. (**B**) UMAP plots derived from integrating scRNA-seq data reported in the HLCA (11) reveal the stromal cell clusters (right panel). (**C**) UMAP visualization (left panels) and violin plots (right panels) compare the mRNA expression levels of *CTHRC1* (as myofibroblast marker), *NPR2* (GC-B), *PKGI* (cGKI), and *PDE2A* in stromal cell clusters from control lungs and lungs from patients with interstitial lung diseases (ILD) or IPF. Significance was tested with the Wilcoxon signed rank test; *P < 0.05.

Supporting the hypothesis that cytokine-driven impairment of the antifibrotic effects of paracrine-acting endogenous CNP contributes to the pathogenesis and progression of lung fibrosis, qPCR studies of human lung tissue samples revealed that the mRNA expression levels of GC-B (mildly) and cGKI are diminished in IPF lungs (Figure 6A). Moreover, the pulmonary expression levels of PDE2A are significantly upregulated (Figure 6A). To determine whether these “global” tissue changes involve alterations within fibroblast populations, we used published scRNA sequencing data sets from the HLCA (11) to compare gene expression levels in the stromal cell clusters between “healthy” control lungs, ILD and IPF. The Uniform Manifold Approximation and Projection (UMAP) plots are shown in Figure 6B. As shown in the UMAP and the violin plots (Figure 6C), *CTHRC1* was significantly upregulated in stromal lung cells from ILD and IPF. Concomitantly the expression levels of *NPR2* (GC-B) and *PKGI* (cGKI) were significantly reduced. In contrast to the observations in whole lung samples (Figure 6A), stromal *PDE2A* expression levels were similar in control and IPF lungs, and they were even reduced in ILD (Figure 6C). This discrepancy warrants further investigation. In future studies, we will try to analyze PDE2A protein expression in specific lung fibroblast subpopulations to unravel potential cell type–specific alterations. However, taken together, these findings indicate that fibroblast CNP/GC-B signaling is altered in inflammation-driven experimental and clinical PF.

### A single s.c. injection of MS-conjugated long-acting CNP prevents bleomycin-induced lung inflammation and fibrosis.

Exogenous high-dose CNP infusions exerted antiinflammatory and antifibrotic actions in LPS-induced acute lung injury and bleomycin-induced PF (6, 7) despite the here-observed receptor and postreceptor changes. Thus, impaired CNP signaling might be overcome by high CNP concentrations. The short 3-minute half-life of CNP and consequently derived need for constant or repeated CNP infusions hamper the translation of such preclinical observations into clinical trials. The stabilized CNP analog vosoritide has recently been approved for treatment of achondroplasia in children (23). However, this requires daily s.c. injections. Recently, a long-acting stabilized CNP analog MS~[Gln^6,14^]CNP-38 was developed, with release rates allowing once-weekly and once-monthly s.c. injections (10), a regimen that would be acceptable in patients with chronic PF. This compound was generated using a tunable, releasable linker system to attach a stabilized CNP analog, [Gln^6,14^]CNP-38, to a long-lived MS carrier. Injection of MS~[Gln^6,14^]CNP-38 creates a s.c. deposit that slowly releases the peptide (10). As proof of principle, first we compared the effects of [Gln^6,14^]CNP-38 and regular, unmodified short-acting CNP (CNP-22) in cultured human lung fibroblasts. As shown in Supplemental Figure 6, A and B, both peptides similarly enhanced intracellular cGMP levels and reduced PDGF-BB–stimulated fibroblast proliferation. Therefore, we tested the MS-conjugated long-acting CNP analog in experimental bleomycin-induced PF.

**Figure 7:**
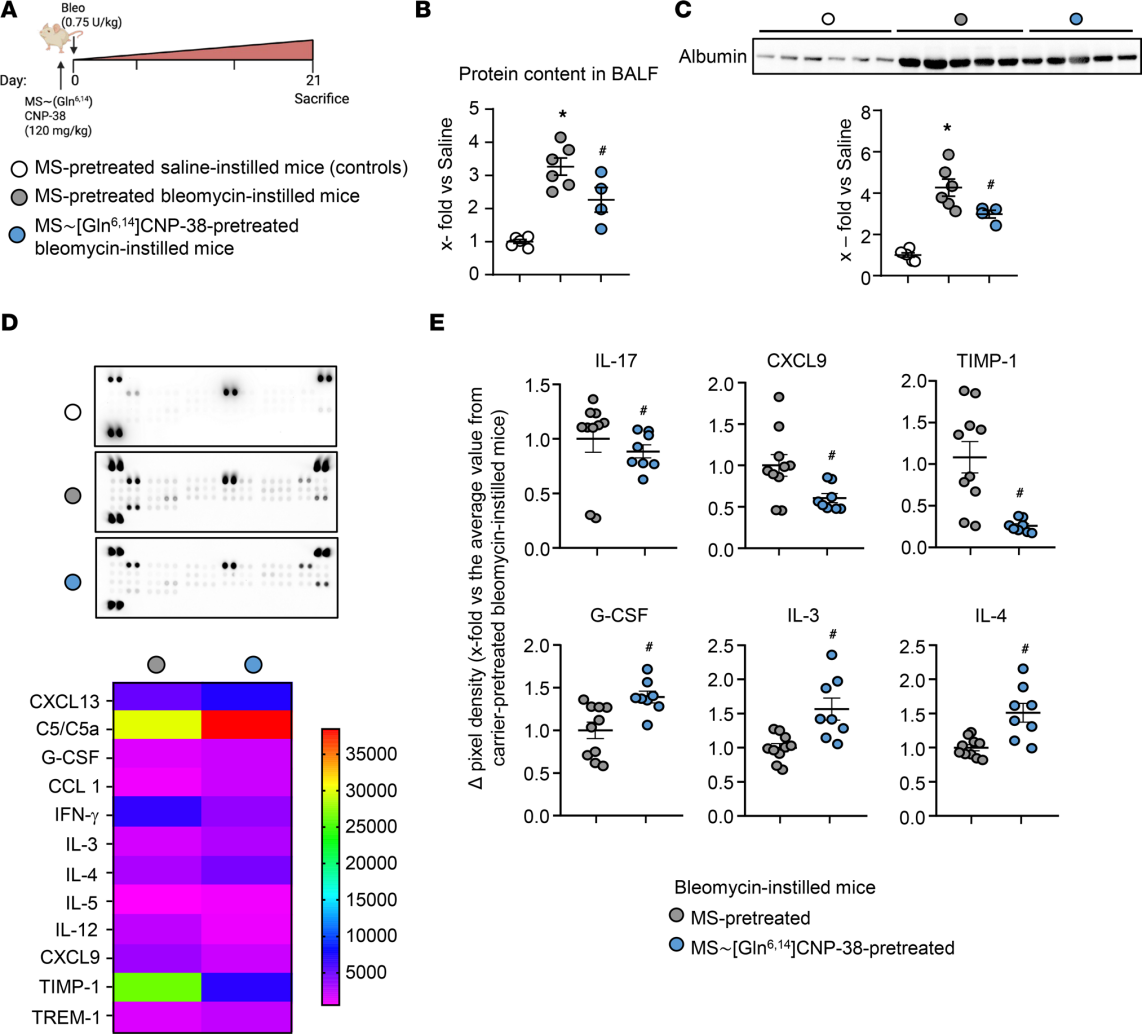
A single subcutaneous injection of long-acting MS conjugated MS~[Gln^6,14^]CNP-38 prevents bleomycin-induced lung inflammation. (**A**) Schematic illustration of these studies performed in control mice 21 days after bleomycin or vehicle (saline) administration. Bleomycin-instilled mice were pretreated with a single s.c. injection of either carrier (empty MS, as vehicle) or MS~[Gln^6,14^]CNP-38. (**B** and **C**) Plasma leakage was evaluated by determination of the protein and albumin contents of bronchoalveolar lavage fluid (BALF) samples by BCA assay (**B**) and immunoblotting (**C**). (**D** and **E**) Cytokine contents of BALF samples were evaluated with a commercial array. **D** shows representative cytokine array membranes probed with BALF from each study group. The heatmap depicts cytokines which were detected at markedly different levels in BALF from carrier (MS)- or MS~[Gln^6,14^]CNP-38–pretreated bleomycin-treated mice (mean-normalized values are shown, with red color indicating high, and blue indicating low values). (**E**) Quantitative evaluation of pixel densities of representative cytokines differing between these 2 groups. In **B** and **C**, each data point represents an individual study mouse; **E** shows replicates from 4–5 mice per group. Significance was tested by 1-way ANOVA (**B** and **C**), and Student’s unpaired *t* test (**D**). *P < 0.05 versus vehicle-instilled mice; ^#^*P* < 0.05 versus carrier-pretreated/bleomycin-treated mice.

Study mice received a single s.c. injection of 120 mg/kg body weight (BW) MS~[Gln^6,14^]CNP-38 (in a volume of 9 mL/g BW) or carrier alone (9 mL/g BW MS, as vehicle) 10 minutes prior to the intratracheal instillation of bleomycin (0.75 IU/kg BW) (Figure 7). Three days later, the CNP plasma levels on average were 31-fold higher in the MS~[Gln^6,14^]CNP-38–treated mice (averaging 90 ng/mL, which corresponds to 41.3 nM) as compared with vehicle-treated mice (2.9 ng/mL corresponding to 1.3 nM; *n* = 4 per condition) The studies of lung morphology and function were performed 21 days after bleomycin treatment, and at this time, the circulating CNP levels were still approximately 4-fold higher in MS~[Gln^6,14^]CNP-38 as compared with vehicle-treated mice, which is consistent with the published pharmacokinetics (10).

Pulmonary inflammation was evaluated by analyses of BALF. Figure 7, B and C, illustrates that bleomycin-evoked plasma protein — i.e., albumin extravasation at this late time point (21 days) — was like that observed 7 days after bleomycin administration (see previous Figure 3, B and C), indicating persistent inflammation. As also shown, this response was significantly prevented by a single s.c. injection of MS~[Gln^6,14^]CNP-38. Further characterization of the BALF with a cytokine array confirmed that numerous proinflammatory cytokines and chemokines were increased in BALF from vehicle (“empty” MS) pretreated bleomycin-instilled mice (Figure 7D), although the pattern at this late time point was different to that observed at earlier time points (Figure 3D). As shown, this response was blunted in the MS~[Gln^6,14^]CNP-38–treated mice. Figure 7E depicts the cytokines that were mostly affected by this treatment. Notably, the BALF levels of proinflammatory cytokines such as IL-17 and CXCL9 (16, 24) were reduced by the CNP analog, whereas the levels of antiinflammatory, antifibrotic factors such as G-CSF, IL-3, and IL-4 (14, 25, 26) were increased. Additionally, [Gln^6,14^]CNP-38 almost abolished the bleomycin-induced increases of tissue inhibitor of metalloproteinase 1 (TIMP-1), which may improve MMP-mediated interstitial collagen degradation (Figure 7E).

**Figure 8:**
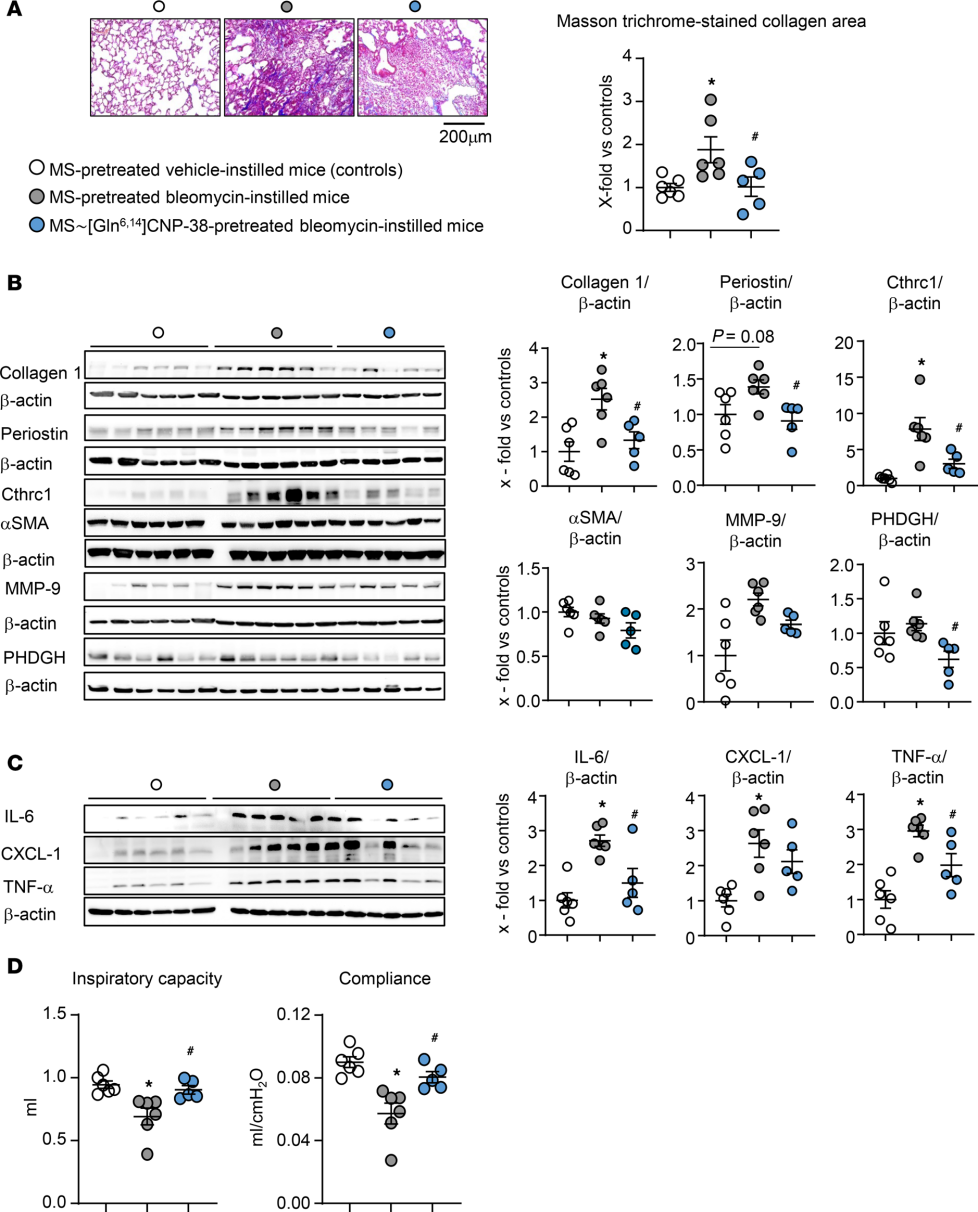
A single s.c. injection of long-acting MS-conjugated MS~[Gln^6,14^]CNP-38 prevents bleomycin-induced lung fibrosis. The animal protocol was illustrated in Figure 7. (**A**) Collagen deposition was determined by Masson’s trichrome staining of lung paraffin sections, followed by quantification with ORBIT. The mean value from 2 sections per mouse was taken (10–15 fields per section were evaluated). Collagen areas (in percent from corresponding total section areas) are presented as x-fold from the average value of control mice. (**B** and **C**) The pulmonary expression levels of fibrosis and myofibroblast markers Collagen 1, Periostin, Cthrc-1, α-SMA, MMP-9, and PHDGH (**B**) as well as of proinflammatory cytokines IL-6, CXCL-1, and TNF-α (**C**) were studied by immunoblotting and normalized to β-actin; values were calculated as x-fold from the average value of control mice. (**D**) Lung inspiratory capacity and compliance were evaluated in anesthetized mice with a Flexivent system. *n* = 6 (Carrier/saline), 6 (Carrier/bleomycin) and 5 mice (MS~[Gln^6,14^]CNP-38/bleomycin). Significance was tested by 1-way ANOVA. *P < 0.05 versus carrier/saline-treated mice; ^#^*P* < 0.05 versus carrier/bleomycin-treated mice.

Indeed, Masson′s trichrome staining of lung sections together with immunoblot analyses of lung samples revealed that bleomycin-induced interstitial collagen accumulation was reduced by MS~[Gln^6,14^]CNP-38 (Figure 8, A and B). Accordingly, in vehicle-pretreated mice, the administration of bleomycin increased the pulmonary expression levels of the myofibroblast markers periostin and Cthrc1, phosphoglycerate dehydrogenase (PHDGH, involved in the early steps of L-serine synthesis for collagen), and MMP-9. Pretreatment of study mice with [Gln^6,14^]CNP-38 abrogated all these changes (Figure 8B). Notably, MS~[Gln^6,14^]CNP-38 also attenuated the bleomycin-induced expression of the proinflammatory cytokines IL-6, TNF-α, and (less) CXCL1 (Figure 8C). Similar to the previous studies (Figure 4C), whole-lung α-SMA levels were not different between bleomycin- and vehicle-treated mice (Figure 8B).

The effect on lung function was evaluated with the Flexivent system (Figure 8D). In vehicle-pretreated mice, bleomycin induced a significant decline of inspiratory capacity and compliance. These functional changes were fully prevented by MS~[Gln^6,14^]CNP-38. Collectively, our results demonstrate that a single dose of this long-acting CNP analog led to long-lasting elevations of plasma CNP levels, which blunted bleomycin-induced lung inflammation, fibrosis, and dysfunction.

### CNP reverts the profibrotic activation of cultured PF fibroblasts in vitro and in situ.

In the previously illustrated experiments in vitro and in vivo, we tested the “preventive” antifibrotic effects of CNP. To further explore the therapeutic potential, lastly, we performed treatment studies in 2 models of cultured human PF fibroblasts. In vitro, cultured IPF lung fibroblasts were prestimulated with TGF-β for 24 hours to induce profibrotic changes, followed by treatment with vehicle (PBS) or CNP (100 nM) for additional 24 hours (Supplemental Figure 7A provides a scheme). TGF-β stimulation significantly upregulated the expression of Collagen 1 and Cthrc 1. Notably, subsequent CNP treatment significantly reduced these changes (Supplemental Figure 7A). In a second experimental series, we studied cultured precision-cut lung slices (PCLS) from a patient with end-stage PF caused by HP (2). As illustrated in Supplemental Figure 7B, the slices were cultured in the presence of CNP (100 nM, daily addition) or vehicle (PBS) for 7 days. At the end of the experiment, the slices were lysed in RIPA buffer and the extracted proteins were analyzed by immunoblotting. Notably, in CNP-treated PCLS, the expression of Collagen 1 and Cthrc 1 was reduced (in comparison with PBS-treated PCLS; see Supplemental Figure 7B). Together, these results indicate that CNP can prevent and even revert the profibrotic phenotype of human lung fibroblasts in vitro and in situ (in PCLS), supporting further studies to investigate its therapeutic potential.

## Discussion

Most published studies of the pathogenic role of fibroblasts in PF focused on the molecular determinants of their profibrotic activation, with the goal to decipher drivers that might be targets for pharmacotherapy. Much less is known about endogenous “brakes” of pathological, especially inflammation-driven, fibroblast activation. This is important because the failure of such brakes likely contributes to the pathogenesis and/or progression of PF; therefore, their pharmacological “reconstitution” or substitution could improve the therapy of patients with PF. Based on preclinical in vitro/in vivo studies performed with exogenous, synthetic CNP (6, 7), the endogenous hormone CNP, which is produced within the lung (27, 28), might be such an endogenous “brake.” If true, then clinically approved drugs inhibiting neprilysin-mediated CNP degradation and thereby augmenting endogenous CNP actions could have beneficial effects in PF. In this study, using a genetic mouse model with fibroblast-restricted deletion of the GC-B receptor, we show that endogenous paracrine CNP, through GC-B/cGMP signaling in fibroblasts, attenuates the release of fibrokines and cytokines. This reduced acute pulmonary vascular leakage and inflammation following injury by bleomycin. However, in persistent inflammation, the transition of fibroblasts to active myofibroblasts is associated with molecular remodeling of the CNP signaling machinery and a loss of the antifibrotic CNP effects. Such failure of the “CNP brake” might contribute to the initiation or progression of inflammation-driven PF. Indeed, restoring this inhibitory brake with a single s.c. injection of the recently developed long-acting CNP analog, MS~[Gln^6,14^]CNP-38 (10) prevented bleomycin-induced lung inflammation, fibrosis, and dysfunction. Our data indicate the pathophysiological role of a loss of endogenous lung fibroblast CNP/GC-B signaling in PF and characterize the protective effect of augmentation of this pathway with a stabilized synthetic CNP analog. Notably, in cultured human lung fibroblasts, [Gln^6,14^]CNP-38 exerted similar antiproliferative effects as regular CNP, supporting the clinical relevance of our preclinical studies. The pharmacokinetic properties of MS~[Gln^6,14^]CNP-38 allow once-weekly and once-monthly s.c. injections (10), a regimen that would be acceptable in patients with chronic PF.

Fibroblasts, particularly when activated to myofibroblasts, are both targets and amplifiers of inflammation (4). Upon activation, they secrete multiple proinflammatory cytokines, chemokines, and fibrokines, including IL-1, IL-6, CXCLs, CCLs, and GM-CSF. These mediators foster the inflammatory process and thereby fibrosis (4, 13–16, 22–24, 29). Our conclusion that CNP inhibits such proinflammatory features of fibroblasts is based on 3 complementary observations: in cultured activated human lung fibroblasts, CNP inhibited the expression of TNF-α and IL-6; bleomycin-treated transgenic mice with fibroblast-restricted inhibition of CNP signaling (Fibro GC-B KO) had increased pulmonary levels of such fibrokines and cytokines, especially of CXCL13, GM-CSF, MCP-1/CCL2, CXCL12, TNF-α, IL-1α, and IL-6, together with enhanced vascular leakiness; and application of the long-acting CNP analog MS~[Gln^6,14^]CNP-38 exerted antiinflammatory effects. Interestingly, along with reduction in proinflammatory cytokines like IL-17 and CXCL9, MS~[Gln^6,14^]CNP-38 treatment increased the levels of G-CSF, IL-3, and IL-4 (Figure 7E). G-CSF might contribute to fibrosis resolution by promoting the homing of bone marrow–derived stem cells (BMSCs) to the lungs via the SDF-1/CXCR4 axis (14). IL-3, once considered proinflammatory, has been shown to support immune regulation through Foxp3^+^ T cell induction in models of autoimmune disease (26). Similarly, IL-4, known for its antiinflammatory and tissue repair functions, may aid in fibrosis resolution, although its effects can be context dependent (25). These findings suggest that the long-acting CNP analog promotes an immunomodulatory state rather than a strictly antiinflammatory response, warranting further investigation.

Our molecular studies of human and murine lung fibroblasts reveal the expression of 2 CNP/cGMP-regulated mediatory proteins (as third messengers): the cGMP-stimulated kinase cGKI, and the cGMP-inhibited cAMP-degrading PDE3A (Figure 5A). Activation of cGKI mediates stimulatory effects of CNP on nuclear FoxO3 signaling (30), which may contribute to the counter regulation of the proliferative effects of PDGF-BB. Moreover, cGKI phosphorylates Smads 2/3, thereby preventing their TGF-β–induced nuclear translocation and enhanced fibroblast collagen expression (31). Additionally, the inhibition of PDE3A, mediating a positive cGMP-to-cAMP crosstalk (5), may participate in the protective fibroblast effects of CNP. Increasing fibroblast cAMP levels through activation of Gs-coupled receptors (by prostacyclin and prostaglandin E_2_) or through inhibition of PDE4-mediated cAMP degradation counteracted lung fibroblast proliferation, migration, collagen secretion, and TGF-β–induced myofibroblast differentiation (32). Activated cAMP-dependent protein kinase (PKA), by phosphorylating cAMP-response-element-binding protein (P-CREB-Ser_133_), interferes with TGF-β–driven Smad3/4-dependent transcriptional activation (32, 33). Notably, PDE4 inhibitors and prostacyclin agonists had positive results in phase II and III clinical trials by stabilizing lung function in patients with IPF (34), which recently led to the approval of the selective PDE4B inhibitor nerandomilast (Jascayd) by the FDA (35). This underscores the potential of targeting cyclic nucleotide signaling in PF and encourages further preclinical studies of CNP in comparison or even in combination with PDE4 inhibitors. In fact, combined activation of cGMP and cAMP in lung fibroblasts was already proposed as a therapeutic concept (33).

Notably, our in vivo/in vitro studies revealed inflammation-induced “remodeling” of the CNP signaling pathway in activated fibroblasts. Within resident lung fibroblasts of mice 3 days after bleomycin application, the expression of the molecules mediating CNP effects was unaltered (GC-B, PDE3A) or only mildly reduced (cGKI). However, subsequent fibroblast-to-myofibroblast transition (7 days after bleomycin application) was accompanied by significant alterations of the CNP signaling machinery, with diminished expression of molecules mediating CNP/cGMP effects (GC-B and cGKI) and enhanced expression of molecules participating in CNP (NPR-C) as well as cGMP/cAMP degradation (PDE2A). Such changes were reproduced in cultured human lung fibroblasts upon exposure to cytokines known to be involved in inflammatory PF, such as TNF-α and TGF-β. Molecular alterations of CNP signaling in inflammation-activated myofibroblasts may impair the antifibrotic effects of paracrine CNP. Remarkably, the inflammation-driven induction of PDE2A was also reported in cultured endothelial cells (36), which additionally may impair the known endothelial barrier protecting effects of CNP (5). Supporting the translational and pathophysiological significance, our qPCR studies showed diminished GC-B and cGKI and increased PDE2 mRNA expression in whole lung tissue samples from patients with IPF. Moreover, scRNA-seq data from the HLCA (11) reveal reduced GC-B and cGKI expression in lung stromal cells of patients with ILD or IPF. Although this was not accompanied by increased PDE2 expression, taken together, these data sets in human samples suggest that inflammation evokes molecular alterations of the fibroblast CNP signaling pathway. In future studies, we will try to validate the scRNA data at the protein and activity level. In this context it is somehow surprising that GC-B expression as well as CNP/cGMP signaling and CNP antifibrotic functions were preserved in cultured fibroblasts derived from patients with IPF (at least for high CNP concentrations). This indicates that culture conditions and repeated passaging alter the molecular and functional properties of native cells including fibroblasts (8), and it emphasizes that in vivo/ex vivo studies are indispensable for making proper conclusions.

Indeed, the link between inflammation-driven loss of fibroblast GC-B expression and loss of antifibrotic CNP functions is suggested by our observations in Fibro GC-B–KO mice. During early stages after bleomycin application, when fibroblast GC-B expression was still preserved in control mice (Figure 5C), such KO mice had exacerbated pulmonary inflammation (as compared with controls), unraveling fibroblast-mediated antiinflammatory effects of local paracrine CNP (Figure 3). However, during later stages after bleomycin application, when fibroblast GC-B expression was reduced in control mice (Figure 5D), PF and dysfunction were similar in both genotypes (Figure 4).

As already mentioned in the introductory section, chronic infusions of synthetic CNP exerted protective actions in rodent models of inflammation-driven PF (6, 7). Hence, pharmacological CNP administration may provide lung CNP levels that are able to effectively diminish inflammation and fibrosis despite altered CNP/cGMP signaling. Nevertheless, the short half-life of CNP and derived need for constant CNP infusions or repeated injections hampered the translation of such preclinical observations into clinical trials. Recently, MS~[Gln^6,14^]CNP-38 was developed, utilizing a releasable linker technology to attach the highly active, stabilized CNP analog, [Gln^6,14^]CNP-38, to a MS carrier with programmed release rates (10). This allows once-weekly and once-monthly s.c. injections (10), a regimen that would be acceptable in patients with chronic PF. A single dose of MS~[Gln^6,14^]CNP-38 led to long-lasting elevations of plasma CNP levels in our study mice, which blunted bleomycin-induced lung inflammation, fibrosis, and dysfunction. Of course, actions of [Gln^6,14^]CNP-38 on other GC-B–expressing cells of the lung — e.g., on endothelial cells and immune cells such as mast cells (5, 37) — likely contribute to the observed attenuation of experimental inflammation and fibrosis. The clinical relevance is supported by the observation that CNP reverted the profibrotic collagen-producing hyperactivity of human lung myofibroblasts in situ, in cultured PCLS from a patient with end-stage HP.

In future studies, we will try to solve the following limitations of our present investigations: (a) Published studies revealed sex differences in vascular and cardiac CNP/GC-B expression and/or signaling (38), which might also be relevant for PF and were not addressed here. (b) The design of our studies of long acting MS~[Gln^6,14^]CNP-38 allows to conclude that this synthetic peptide prevented bleomycin-driven lung inflammation and fibrosis; however, for assessing clinical relevance, we must next test whether administration of this peptide during the inflammatory or the fibrotic phase is also able to prevent or revert fibrosis. (c) With the same aim, such studies should include other preclinical models of lung inflammation and fibrosis. (d) Although published scRNA-seq data from the HLCA (11) suggest that the observed inflammation-driven inhibition of the fibroblast CNP/GC-B/cGKI signaling pathway also occurs in diseased human lungs, this pathophysiologically relevant possibility needs further investigations. The here-reported observations provide a basis for obtaining deeper insights into the regulation and pathological dysregulation of pulmonary CNP signaling and its therapeutic targeting.

In summary, here we used a genetic mouse model and a recently developed stabilized long acting CNP analog to dissect the pathophysiological and therapeutic implications of CNP/cyclic GMP signaling in inflammation-driven PF. Our observations indicate that molecular remodeling of activated lung myofibroblasts leads to the loss of an endogenous paracrine antifibrotic “CNP brake,” which may contribute to the progression of this severe disease. Rescuing this pathway with long-acting CNP analogs (10, 39) has therapeutic potential.

## Methods

### Sex as a biological variable.

Our study examined human lung fibroblasts of both male and female patients. The mouse studies also involved both male and female mice.

### Animal studies.

Details of the generation of Fibro GC-B–KO mice were previously published (8). Mice with a floxed GC-B gene (*Npr2^fl/fl^*) were crossed with the *Col1a2-Cre^ERT2^* line. Genotyping was performed using genomic DNA extracted from tissue obtained during ear markings. Four-week-old *Col1a2-Cre^ERT2+/0^ Npr2^fl/fl^* and control littermates lacking the *Cre* transgene (*Npr2^fl/fl^*) were treated for 5 days with daily i.p. injections of tamoxifen (1 mg/day) (8). Mice homozygous for floxed *Npr2* and heterozygous for the *Cre* transgene were studied. *Npr2^fl/fl^* littermates without *Cre^tg^* served as controls within each experiment. The following studies were performed with 8- to 10-week-old littermate mice of mixed (C57BL/6, 129Sv) background.

PF was induced in mouse lungs by a single intratracheal bleomycin administration (0.75 U/kg; Medac GmbH), under 2% isoflurane anesthesia. Sham mice received saline. The depth of the anesthesia was checked by ensuring that noxious pinch stimulation of the hind paw, the forepaw, and the ear did not evoke any motor reflexes. Buprenorphine (0.05–0.1 mg/kg BW) was used for preemptive and post-treatment analgesia.

To assess the effect of long-acting CNP on PF, control mice received a single s.c. injection of 120 mg/kg BW MS-conjugated MS~[Gln^6,14^]CNP-38 (in a volume of 9 mL/g BW) (10) or carrier alone (9 mL/g BW MS) (10) 10 minutes prior to the administration of bleomycin. For studies of CNP plasma levels, a subgroup of vehicle- or MS~[Gln^6,14^]CNP-38-treated mice was euthanized 3 days after the s.c. injection, and CNP plasma levels were determined by a sensitive ELISA (CNP-22 EIA; Phoenix Pharmaceuticals). MS~[Gln^6,14^]CNP-38 was provided by Daniel V. Santi and Eric L. Schneider (ProLynx, Inc.).

For studies of lung inflammation and of the molecular signatures of resident lung fibroblasts, 3 or 7 days after bleomycin administration, the study mice were euthanized under deep anesthesia for collecting BALF and isolating resident lung fibroblasts (see below). To collect BALF, the trachea was cannulated with a 19G needle, and lung tissue was thoroughly irrigated with 1 mL precooled saline. The BALF was centrifuged (3,000*g*, 10 minutes, 4°C), and the supernatant was stored at –80°C. BALF protein concentration was determined by using the BCA method. The levels of albumin and the macrophage marker Mac-2 were determined by immunoblotting. Cytokine levels were determined with a cytokine array (ARY006; R&D Systems).

Terminal closed-chest studies of lung function were performed in ketamine/xylazine-anesthetized (100 mg/kg BW and 20 mg/kg BW, respectively) mice 21 days after bleomycin treatment with a Flexivent system (Scireq, Canada). Mice were mechanically ventilated at a rate of 150 breaths/min, tidal volume of 10 mL/kg, and a positive end-expiratory pressure (PEEP) of 3 cm H_2_O for 3 minutes. Deep inflation perturbation (slow inflation of the lung to 30 cm H_2_O) was initiated to measure the inspiratory capacity. Calibrated pressure-volume loops were recorded to measure compliance. Mice were then sacrificed under deep anesthesia. Lungs were perfused with cold PBS, and the left lung lobes were fixed in 4% buffered formaldehyde and paraffin-embedded for Masson’s trichrome stainings (ab150686, Abcam), followed by collagen imaging with a Leica DMi1 bright-field microscope (Leica). The right lobes were frozen in liquid nitrogen (for protein extraction and immunoblotting).

### Experiments with cultured human and murine lung fibroblasts as well as with native murine lung fibroblasts.

Primary human lung fibroblasts were isolated from explanted IPF lungs (*n* = 10) or nonutilized donor lungs (*n* = 12) using an outgrowth-technique as published (40). Fibroblasts were grown in Fibroblast Growth Medium (MCDB131, Gibco Cell Culture) supplemented with 5% FCS, 1% penicillin-streptomycin (Pen/Strep), 1% L-Glutamine, 0.2% EGF (AF-100-15; Gibco), 0.2% FGF (100-18B; Preprotech), and 0.5% insulin (I9278; Sigma-Aldrich GmbH). Experiments were performed with cells at passages 4 and 5 starved in serum-free medium. To obtain native or cultured murine lung fibroblasts, mice were sacrificed under deep anesthesia, the lungs were digested with Dispase (Sigma-Aldrich GmbH), and the fibroblasts were enriched with magnetic beads coated with antibodies against the fibroblast protein PDGFR-α. As indicated, the cells obtained from mice 3 and 7 days after bleomycin treatment were immediately lysed with RIPA buffer for immunoblot studies. For the functional characterization, fibroblasts from control and KO lungs were cultured in DMEM medium (Gibco) with 10% FCS for 5 days and then passaged twice for the following experiments.

Cell proliferation was monitored by BrdU incorporation (Roche Diagnostics GmbH). Briefly, 8,000 lung fibroblasts were seeded per well and allowed to proliferate for 24 hours. Cells were serum starved for 24 hours, before being pretreated with vehicle or CNP (Bachem) for 30 minutes followed by PDGF-BB stimulation (Preprotech). Proliferation was measured after 24 hours. Data were normalized to vehicle-treated (PBS) cells.

For the migration assay, fibroblasts were grown in 24-well plates until confluent. After 24 hours serum starvation, plates were scratched, washed, and then treated for 30 minutes with CNP or vehicle and thereafter with PDGF-BB or vehicle for 24 hours. Images were captured directly after scratching and 4, 8, and 24 hours later with an inverted light microscope (4× objective, Olympus) to quantify the wound closure (in percentage of the initial scratch area).

For cGMP determinations, the cells were incubated at 37°C with the PDE inhibitor 3-isobutyl-1-methylxanthine (0.5 mM IBMX; Sigma-Aldrich GmbH) for 15 minutes and thereafter with synthetic CNP or vehicle (saline) for an additional 15 minutes (8, 30).

### Immunoblotting.

Cultured fibroblasts and frozen lung samples were lysed with RIPA buffer of the following composition: 25 mM Tris-HCl, pH 7.6, 150 mM NaCl, 1% NP-40, 1% sodium deoxycholate, and 0.1% SDS (Thermo Fisher Scientific). BALF samples were directly mixed with Laemmli buffer. SDS-PAGE and immunoblotting were performed as described previously (8, 30). The sources and dilutions of all used primary antibodies are depicted in Supplemental Table 1. Protein bands were visualized with enhanced chemiluminescence and quantified by densitometry.

### Studies of gene expression in human lung samples.

Total RNA extraction and qPCR were performed as described using SYBR green master mix (A25742, Applied Biosystems) (8). Porphobilinogen deaminase (PBGD) was used as a reference gene. Data are presented as expression levels relative to PBGD using the 2^−ΔCT^ method. The sequences of the primers and probes are listed in Supplemental Table 2.

### scRNA-seq data analysis.

scRNA-seq data were extracted from the Human Lung Cell Atlas (HLCA; https://cellxgene.cziscience.com/collections/6f6d381a-7701-4781-935c-db10d30de293) (11). The complete AnnData object was accessed, and samples were subset to include only 3 categories: IPF, ILD, and healthy controls. From this subset, the data from the stromal cell populations were extracted for focused analysis. The filtered dataset was converted from Scanpy (.h5ad) format into a Seurat object to enable downstream processing in R. All subsequent analyses including differential expression of target genes were performed using the SeuratExtend package (41).

### Precision cut lung slices.

PCLS were generated from an explanted lung of a patient with PF due to HP (2). A lobe was filled with prewarmed agarose solution (2.5% low-melting agarose in DMEM/F12 medium with 10% FCS, 1% Pen/Strep and 250 ng/mL Amphotericin B) via a cannulated bronchus. After solidification of agarose, the lobe was sectioned and punches with a diameter of 1 cm were obtained. PCLS (300 μm thick) were obtained by slicing the tissue punches with a vibratome and were cultured in RPMI medium supplemented with 10% FCS, 2 mM Glutamine and 1% Pen/Strep. PCLS were treated with 100 nM CNP or vehicle (saline) daily over 7 days and then lysed with RIPA for protein extraction.

### Statistics.

Statistical analysis was performed using GraphPad Prism, version 9.1.0 (GraphPad Software). Experimental data are presented as the mean ± SEM. Data were tested for normality (Shapiro-Wilk test) and were analyzed for statistical significance by 1-way or 2-way ANOVA or unpaired 2-tailed *t* test, as indicated in the legends to Figures. A *P* value less than 0.05 was considered significant.

### Study approval.

All human lung tissue samples were collected in frame of the European IPF registry (eurIPFreg) and provided by the UGMLC Giessen Biobank (member of the DZL Platform Biobanking). The collection of biospecimen (including PCLS) was approved by the Ethics Committee of the Justus-Liebig-University Giessen (No. 111/08 and 58/15), and written informed consent was received from each patient. The studies of cultured human lung fibroblasts and human lung tissues were approved by the ethics committees of Würzburg (approval no. 20220831 02).

The animal studies were approved by the local government (Regierung von Unterfranken, approval nos. 55.2 2532-2-1302-23 and -1302-48) and conformed to the Animal Research: Reporting of In Vivo Experiments (ARRIVE) guidelines.

### Data availability.

Values for all data points in graphs can be found in the Supporting Data Values file. Details on the antibodies and primers used in this study are provided in the Supplemental Tables 1 and 2.

## Author contributions

SD and MK designed the experiments. Experiments were performed by RW, KV, MA, LK, ALF, EL, TP, DS, AB, and SD. Data were analyzed by RW and SD. qPCR and HLCA analyses were carried out by AK and EEA. Patient-derived normal and IPF fibroblasts and human lung tissues were provided by CR. MS~[Gln^6,14^]CNP-38 and [Gln^6,14^]CNP-38 were provided by DVS and ELS. The manuscript was written by SD and MK.

## Funding support

- Deutsche Forschungsgemeinschaft (KU 1037/13-1, KU 1037/16-1, and SFB 1525 to MK; DFG DA 2462/1-1 and DA 2462/3-1 to SD)
- Else-Kröner-Fresenius Stiftung (2020 EKEA.131 to SD).

## Supplementary Material

Supplemental data

Unedited blot and gel images

Supporting data values

## Version 1 01/07/2026

In-Press Preview

## Version 2 02/23/2026

Electronic publication
