# Supplementary material for: Pathophysiological and therapeutic implications of C-type natriuretic peptide/cyclic GMP signaling in pulmonary fibrosis
Source: JCI Insight. 2026 Jan 6;11(4):e196812. doi: 10.1172/jci.insight.196812 (PMC12956015; doi:10.1172/jci.insight.196812)
Supplement: Supplemental data [file jciinsight-11-196812-s137.pdf]

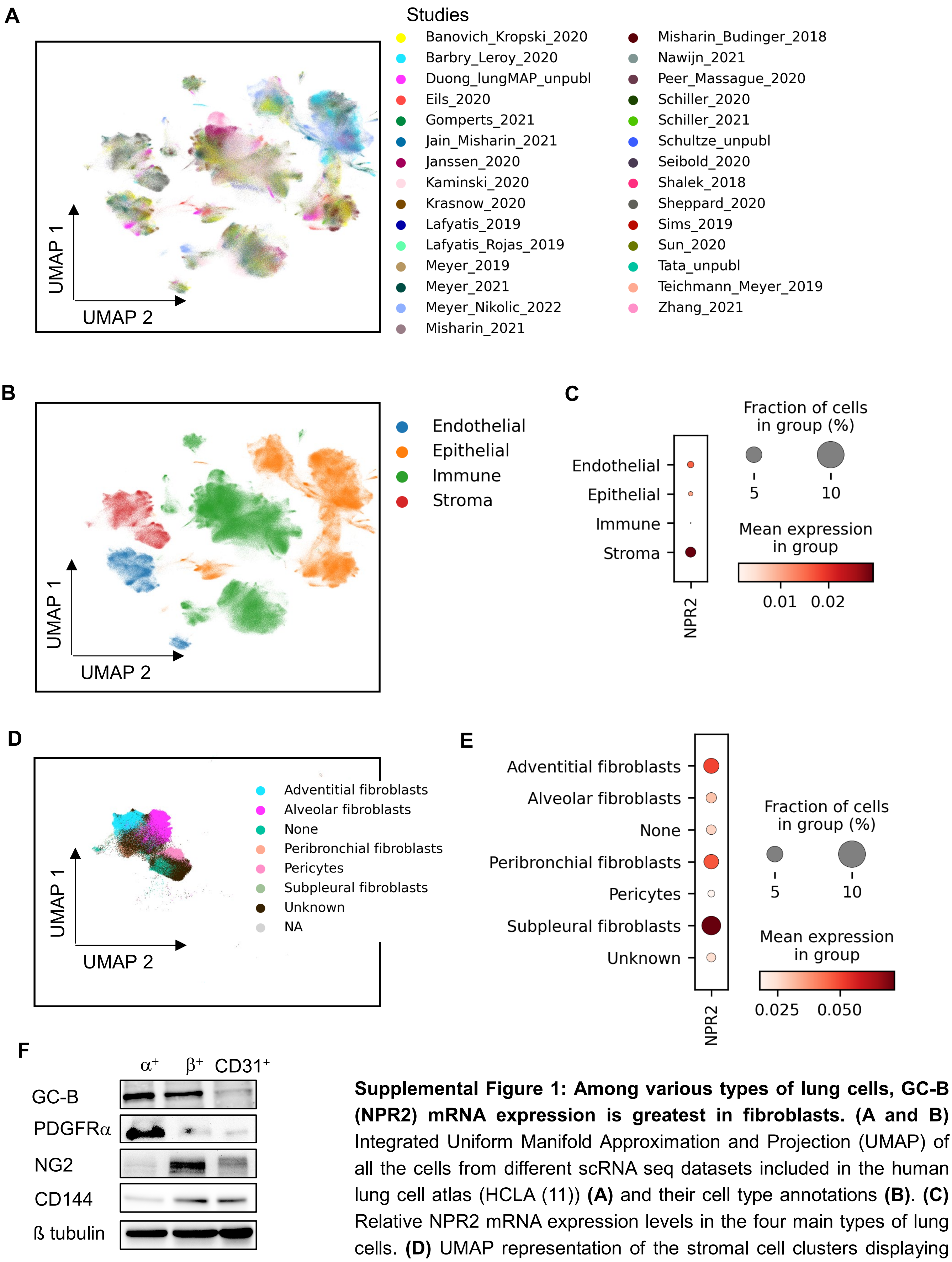


**Supplemental Figure 1: Among various types of lung cells, GC-B (NPR2) mRNA expression is greatest in fibroblasts. (A and B)** Integrated Uniform Manifold Approximation and Projection (UMAP) of all the cells from different scRNA seq datasets included in the human lung cell atlas (HCLA (11)) **(A)** and their cell type annotations **(B)**. **(C)** Relative NPR2 mRNA expression levels in the four main types of lung cells. **(D)** UMAP representation of the stromal cell clusters displaying distinct fibroblast subtypes**. (E)** Dot plot illustrating NPR2 expression across these stromal cell clusters. For **C and E**, dot sizes represent the fraction of cells expressing NPR2 and color intensity represents mean expression. **(F)** Dispase-digested murine lungs (n = 2) were used to separate and enrich PDGFRα$^+$ fibroblasts, PDGFRβ$^+$ smooth muscle cells/pericytes and CD31$^+$ endothelial cells. GC-B, PDGFRα, NG2 and CD144/VE-cadherin expression in these three fractions was studied by immunoblotting.

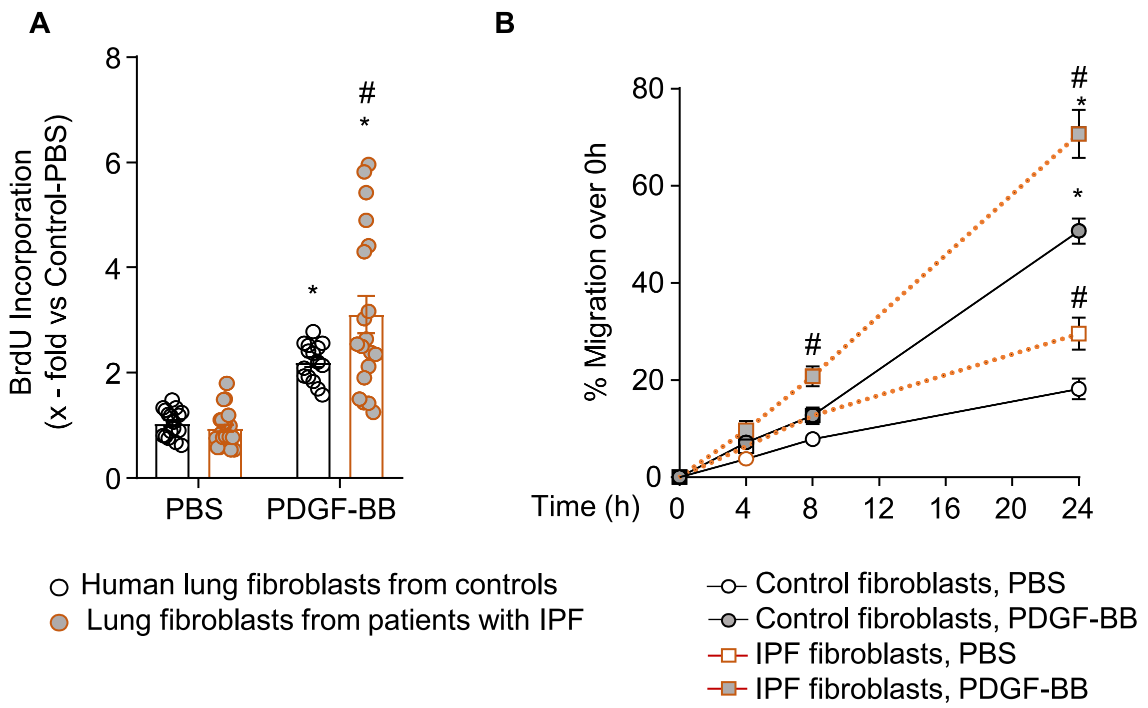


**Supplemental Figure 2: Cultured lung fibroblasts from patients with IPF retain features of hyperactivity, with increased proliferation and migration.** Baseline and PDGF-BB (50 ng/ml)-induced proliferation **(A)** and migration **(B)** of control and IPF fibroblasts was studied at passages 3 - 5, using BrdU incorporation and scratch assays, respectively. The progressive closing of the scratch-induced wound area was quantified 4, 8 and 24 hours after the scratch and calculated as percent of the initial wound area. n=12-16 wells from 3 biological replicates per group. *$p$<0.05 vs PBS; #$p$<0.05 vs control fibroblasts tested by 2-way ANOVA.

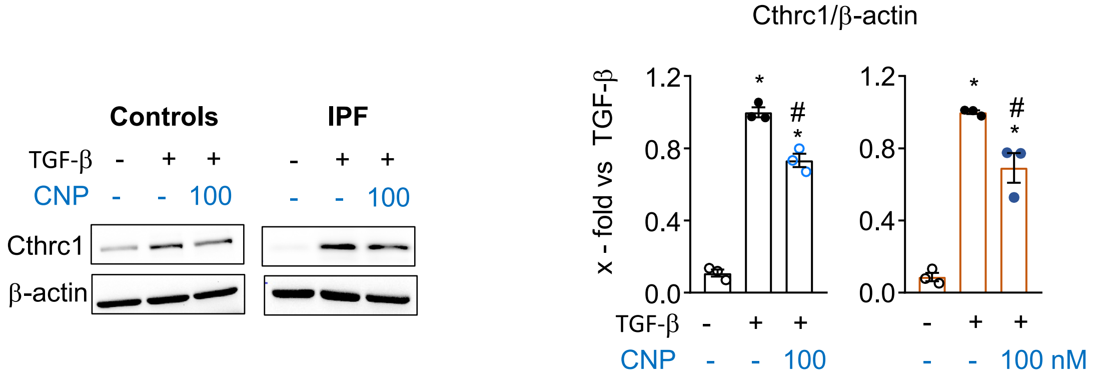


**Supplemental Figure 3: In cultured human lung fibroblasts CNP attenuates the stimulatory effect of TGF-β on Cthrc1 expression; this inhibitory effect is preserved in IPF fibroblasts**. The sample number is indicated by the number of data points in each histogram. Significance was determined by 1-way ANOVA. *$p$<0.05 vs PBS (-/-), #$p$<0.05 vs TGF-β.

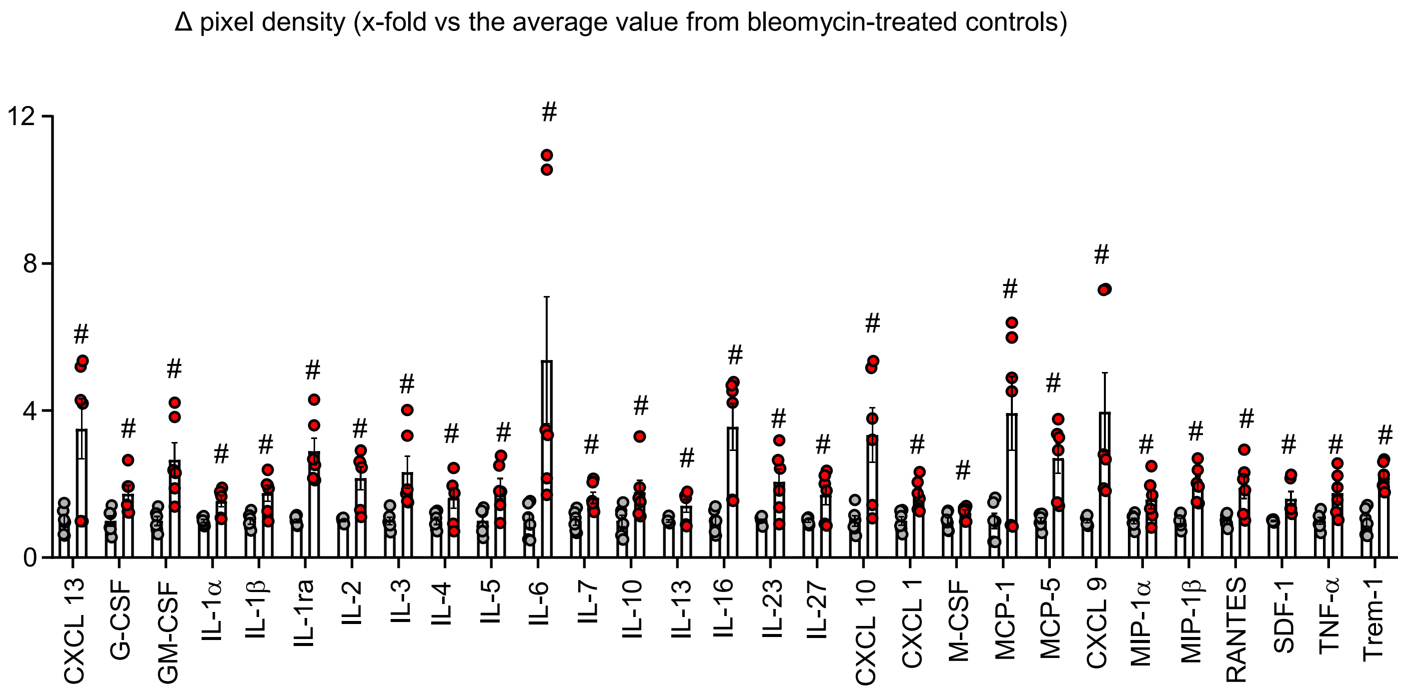


**Supplemental Figure 4**: **Fibroblast-restricted GC-B deletion led to increased cytokine levels in BALF.** Quantitative evaluation of all the cytokines depicting significant differences between bleomycin-treated control and KO mice. Graph shows replicates from 3 mice per group. Significance was tested by Student's unpaired t-test, $^{\#}p<0.05$ vs control mice.

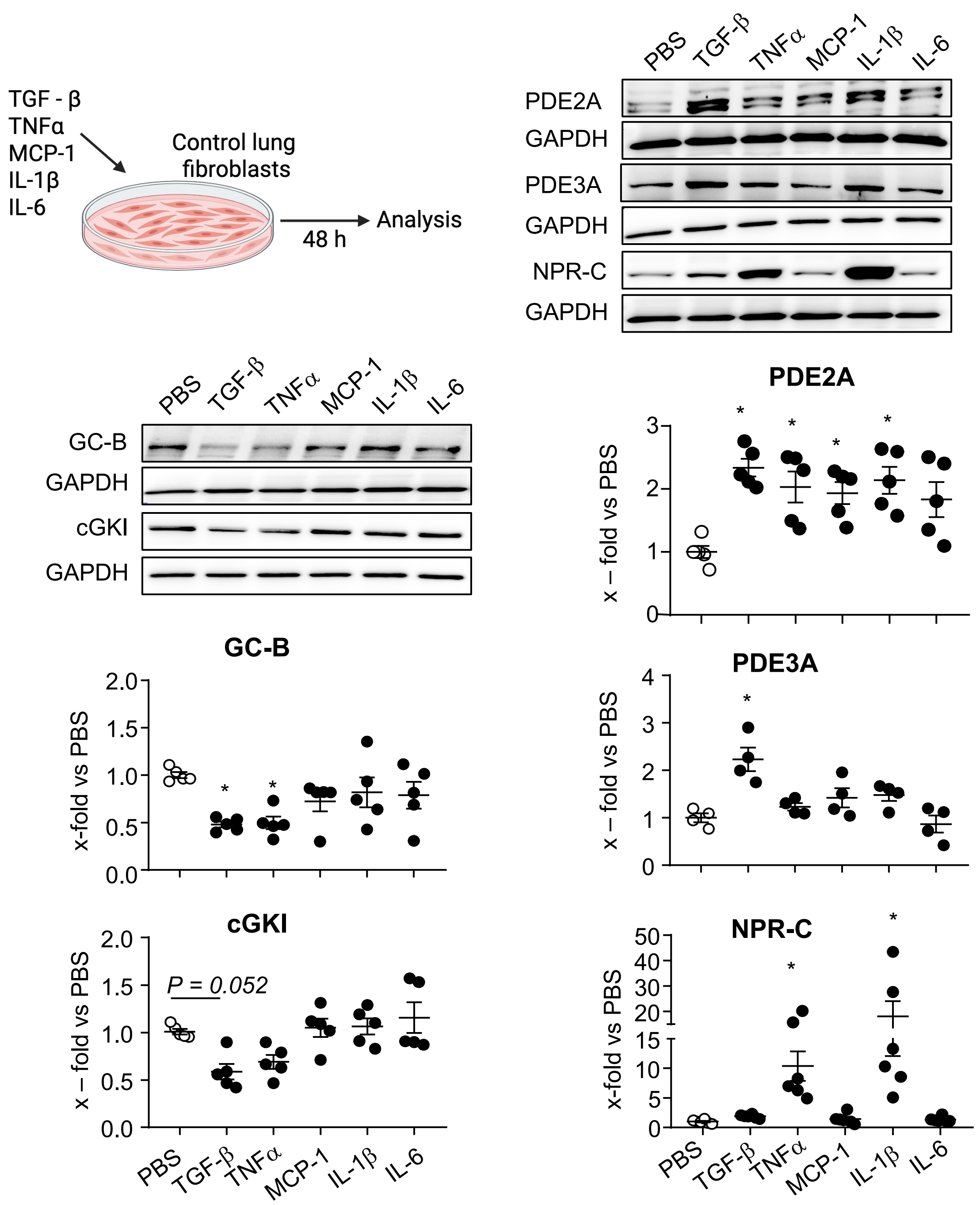


**Supplemental Figure 5: Altered expression of components of the CNP/GC-B signaling pathway in cytokine-treated cultured human lung fibroblasts**. The effect of different cytokines (10 ng/ml of TGF-β, TNFα, MCP-1 or IL-1β; 50 IU/ml of IL-6; all 48 h) on the expression of αSMA, GC-B, cGKI, PDE2A, PDE3A and NPR-C in cultured human lung fibroblasts was analysed by immunoblotting. The sample number for each experiment (n) varied between 4 and 6. Significance was tested by 1-way. *p<0.05 vs PBS.

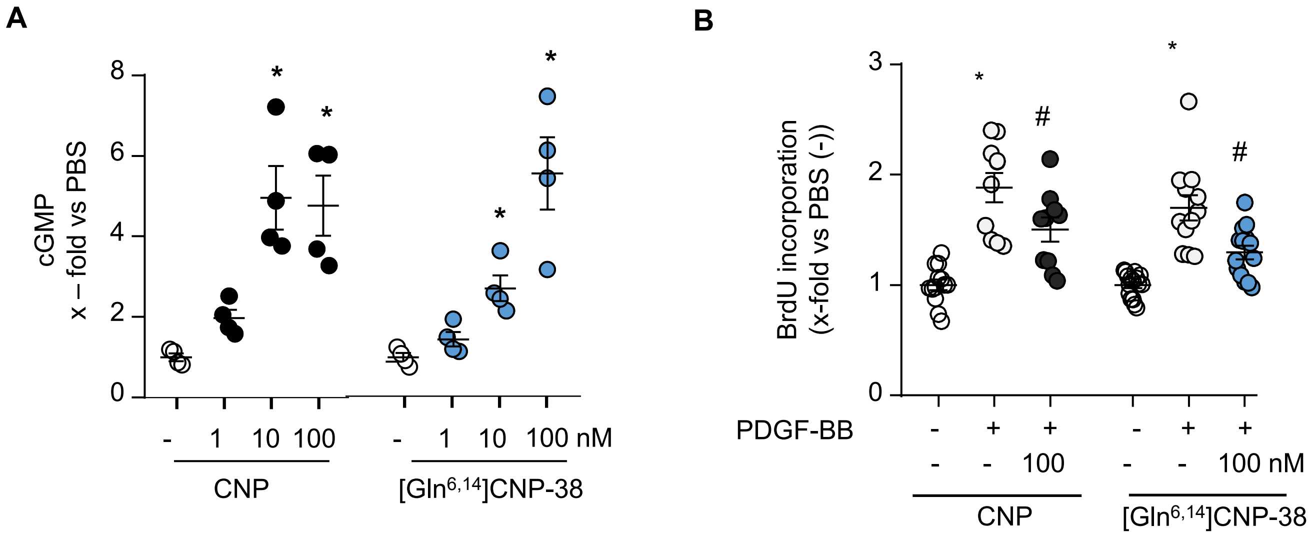


**Supplemental Figure 6: The stabilized CNP-analog, [Gln$^{6,14}$]CNP-38, and regular, unmodified CNP (CNP-22) have similar effects on cultured human lung fibroblasts. (A)** Effects of [Gln$^{6,14}$]CNP-38 and CNP (1-100 nM, 10 min) on intracellular cGMP contents of control lung fibroblasts (n=4 wells from 2 biological replicates in each group)); **(B)** Effects of [Gln$^{6,14}$]CNP-38 or CNP pretreatment (100 nM for 30 min) on PDGF-BB (50 ng/ml, 24h)-induced proliferation (BrdU incorporation was studied in 9-12 wells from 3 biological replicates per group). Significance was determined by 1-way ANOVA. $^{*}p<0.05$ vs PBS (-/-), $^{\#}p<0.05$ vs PDGF-BB.

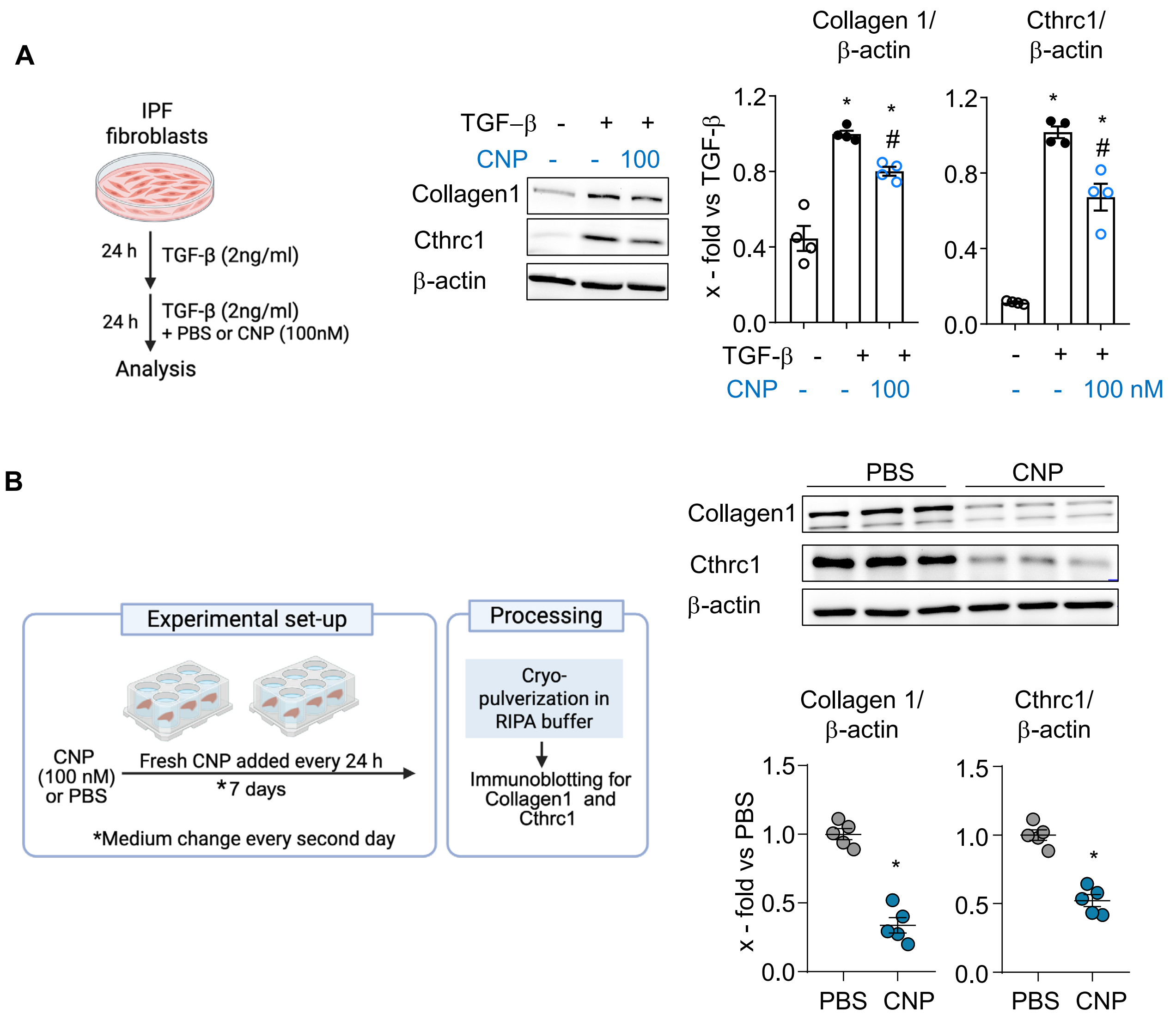


**Supplemental Figure 7: CNP reverts the profibrotic activation of cultured human lung fibroblasts *in vitro* and *in situ*. (A)** Left panel: Schematic representation of the experiment with cultured lung fibroblasts from IPF patients. Right panel: Immunoblot studies demonstrated that TGF-β enhanced collagen 1 and cthrc 1 expression in IPF fibroblasts and that subsequent addition of CNP reverted this effect. The sample number is indicated by the number of data points in each histogram. **(B)** Left panel: Illustration of the studies of cultured PCLS from a patient with PF provoked by hypersensitivity pneumonitis (HP). Right panel: Effect of CNP treatment (7 days) on collagen 1 and cthrc1 expression in such PCLS. The data points indicate the number of slices. Significance was tested by 1-way ANOVA (A) and Student's unpaired t-test (B). *$p<0.05$ vs PBS (-/-), $^{\#}p<0.05$ vs TGF-β.

**Supplemental Table 1**

**Antibodies used for Immunoblotting**

| Target antigen | Vendor or Source | Catalog # | Working dilution |
|---|---|---|---|
| **Albumin** | Biotechne/R&D Systems, Minneapolis, USA | AF3329 | 1:1000 |
| **Biotin-labelled anti-PDGFR$\alpha$** | Miltenyi biotec, Cologne, Germany | 130-101-905 | 1:1000 |
| **cGK I** | Cell Signaling, Leiden, Netherlands | 3248S | 1:1000 |
| **Collagen 1 (Human)** | Proteintech, Planegg-Martinsried, Germany | 14695-1-AP | 1:1000 |
| **Collagen 1 (Mouse)** | Meridian life science, Memphis, USA | T40777R | 1:1000 |
| **Cthrc-1** | Proteintech, Planegg-Martinsried, Germany | 16534-1-AP | 1:1000 |
| **CXCL-1** | Biotechne/R&D Systems, Minneapolis, USA | AF-453-SP | 1:1000 |
| **GAPDH** | Proteintech, Planegg-Martinsried, Germany | 10494-1-AP | 1:5000 |
| **GC-B** | Prof. Hannes, Schmidt; Tübingen, Germany | Used in previous publication (1) | 1:1000 |
| **IL-6** | Proteintech, Planegg-Martinsried, Germany | 21865-1-AP | 1:1000 |
| **Mac-2/galectin-3** | Cederlane, Burlington, Canada | CL8942AP | 1:1000 |
| **MMP-9** | Proteintech, Planegg-Martinsried, Germany | 10375-2-A | 1:1000 |
| **NPR-C** | OriGene, Herford, Germany | TA501080 | 1:1000 |
| **PDE2A** | FabGennix, Frisco, USA | PD2A-101A | 1:1000 |
| **PDE3A** | Prof. Dr. Viacheslav O. Nikolaev, Hamburg, Germany | Used in previous publication (2) | 1:1000 |
| **Periostin** | Novus Biologicals, Wiesbaden Nordenstadt, Germany | NBP1-30042 | 1:1000 |
| **Peroxidase-conjugated Goat anti-guinea pig IgG** | Jackson Immunoresearch, West grove, USA | 106-035-003 | 1:5000 |

| | | | |
|---|---|---|---|
| **Peroxidase-conjugated Goat anti-mouse IgG** | Jackson Immunoresearch, West grove, USA | 115-035-1062 | 1:5000 |
| **Peroxidase-conjugated Goat anti-rabbit IgG** | Jackson Immunoresearch, West grove, USA | 111-035-144 | 1:5000 |
| **PHGDH** | Santa Cruz, Heidelberg, Germany | sc-100317 | 1:1000 |
| **TNFα** | Proteintech, Planegg-Martinsried, Germany | 17590-1-AP | 1:1000 |
| **β-Actin** | Cell signaling, Leiden, Netherlands | 4970S | 1:5000 |
| **β-Tubulin** | Proteintech, Planegg-Martinsried, Germany | 10094-1-AP | 1:5000 |
| **αSMA** | Sigma-Aldrich GmbH, Steinheim, Germany | A5228 | 1:10000 |

## Major Resources Table 2

**Primers used for qRT PCRs**

| **Gene Name** | **Forward primer** | **Reverse Primer** |
|---|---|---|
| **NPR2** | TGTGTATATCTGCGGCCCTC | CGGGCTCTTATCAGCAGACG |
| **PKGI** | GATACCCTTGGAGTTGGAGGT | ATGTGCTCCTGCTGTCTTGT |
| **PDE2A** | CCAGCTGGTGTGTGAGGAC | CGCTTCTGGGAGAT ATAGC |

**References:**


1. Dabral S et al. C-type natriuretic peptide/cGMP/FoxO3 signaling attenuates hyperproliferation of pericytes from patients with pulmonary arterial hypertension. *Commun Biol.* 2024;7(1):693.
2. Špiranec Spes K et al. Heart-Microcirculation Connection: Effects of ANP (Atrial Natriuretic Peptide) on Pericytes Participate in the Acute and Chronic Regulation of Arterial Blood Pressure. Hypertension. 2020 Nov;76(5):1637-1648.
