# Supplementary material for: Pathophysiological and therapeutic implications of C-type natriuretic peptide/cyclic GMP signaling in pulmonary fibrosis
Source: JCI Insight. 2026 Jan 6;11(4):e196812. doi: 10.1172/jci.insight.196812 (PMC12956015; doi:10.1172/jci.insight.196812)

Uncropped blots

Figure 1A

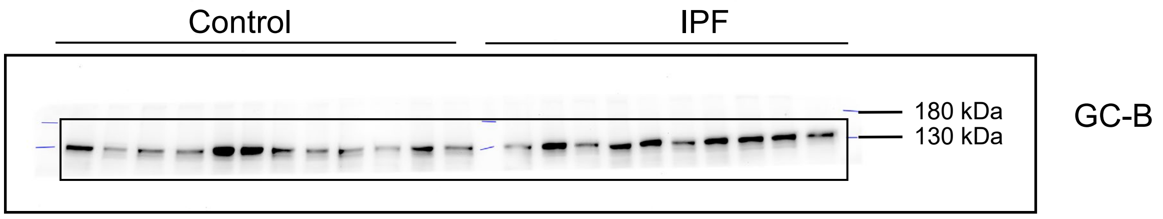

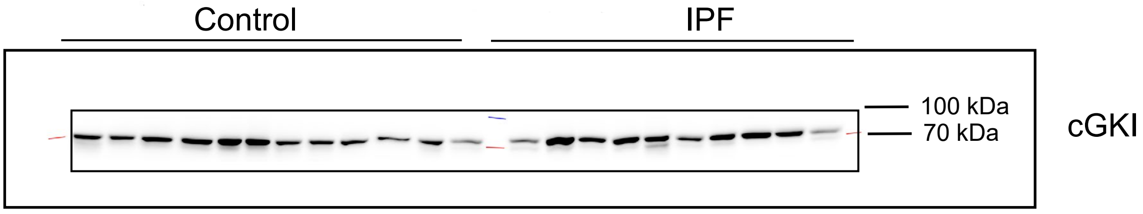

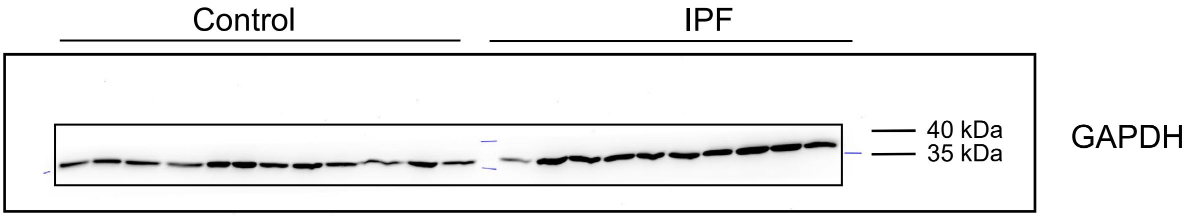

Figure 1E

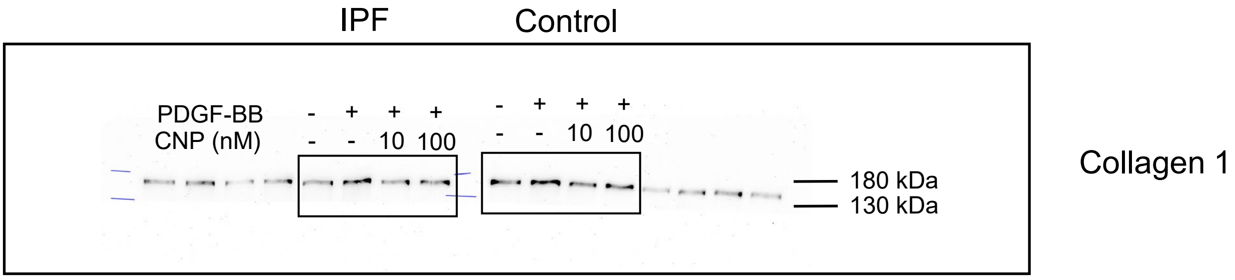

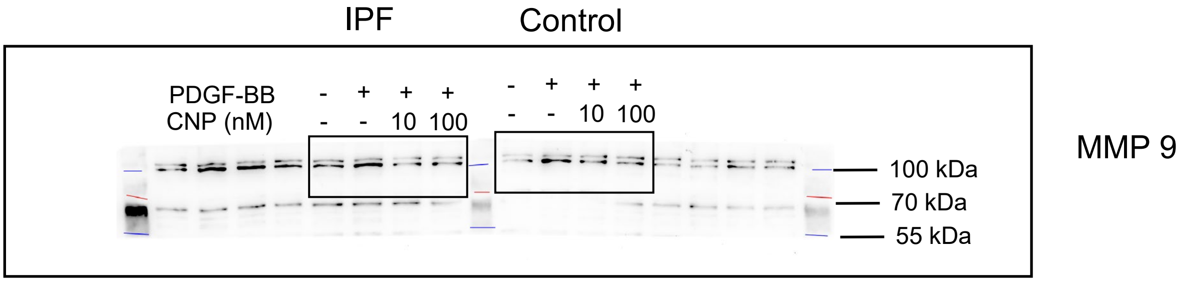

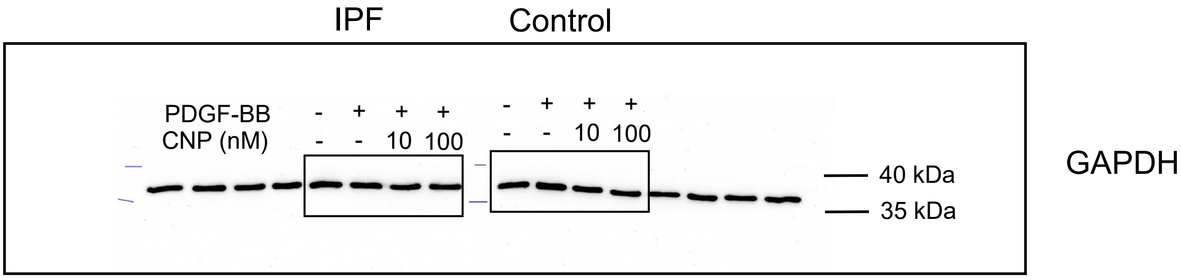

Figure 1F

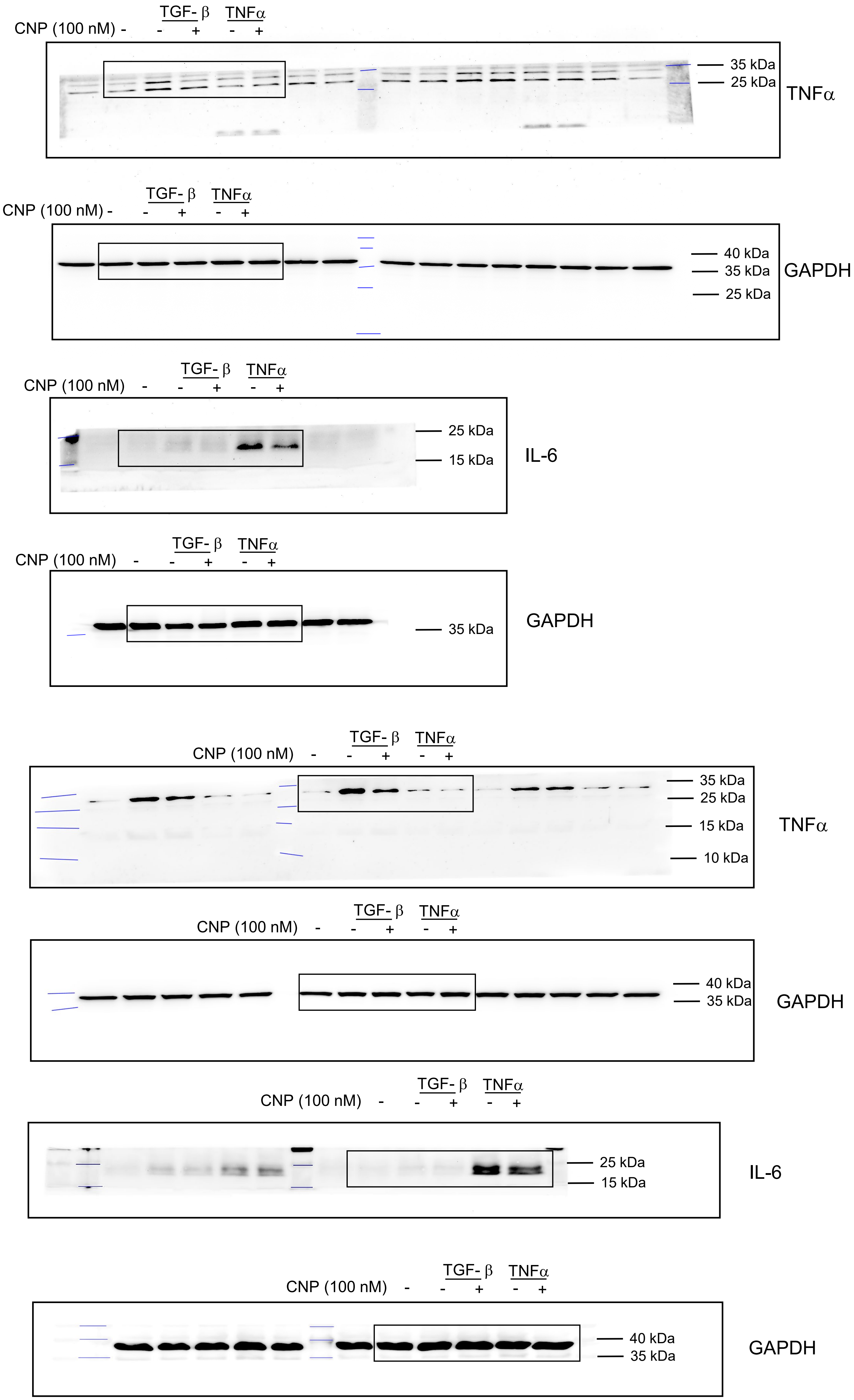

TGF- β
TNFα
CNP (100 nM) - - + - +
35 kDa
25 kDa
TNFα
TGF- β
TNFα
CNP (100 nM) - - + - +
40 kDa
35 kDa
25 kDa
GAPDH
TGF- β
TNFα
CNP (100 nM) - - + - +
25 kDa
15 kDa
IL-6
TGF- β
TNFα
CNP (100 nM) - - + - +
35 kDa
GAPDH
TGF- β
TNFα
CNP (100 nM) - - + - +
35 kDa
25 kDa
15 kDa
10 kDa
TNFα
TGF- β
TNFα
CNP (100 nM) - - + - +
40 kDa
35 kDa
GAPDH
TGF- β
TNFα
CNP (100 nM) - - + - +
25 kDa
15 kDa
IL-6
TGF- β
TNFα
CNP (100 nM) - - + - +
40 kDa
35 kDa
GAPDH

Figure 2A

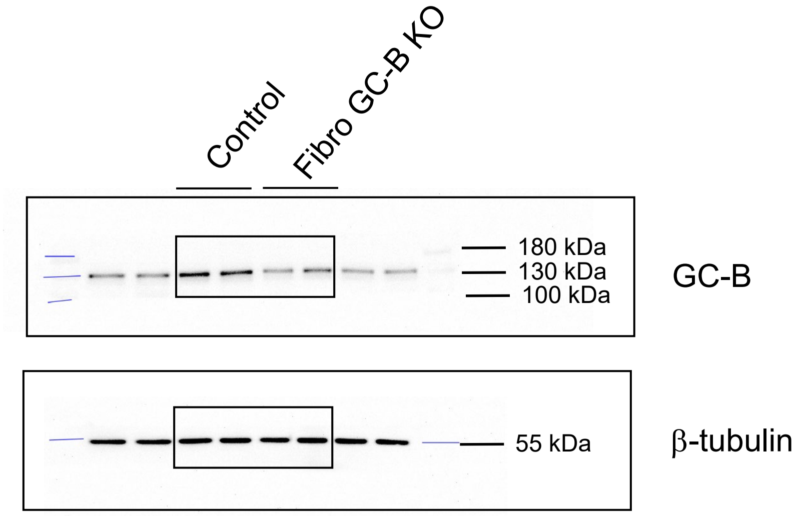

Figure 2E

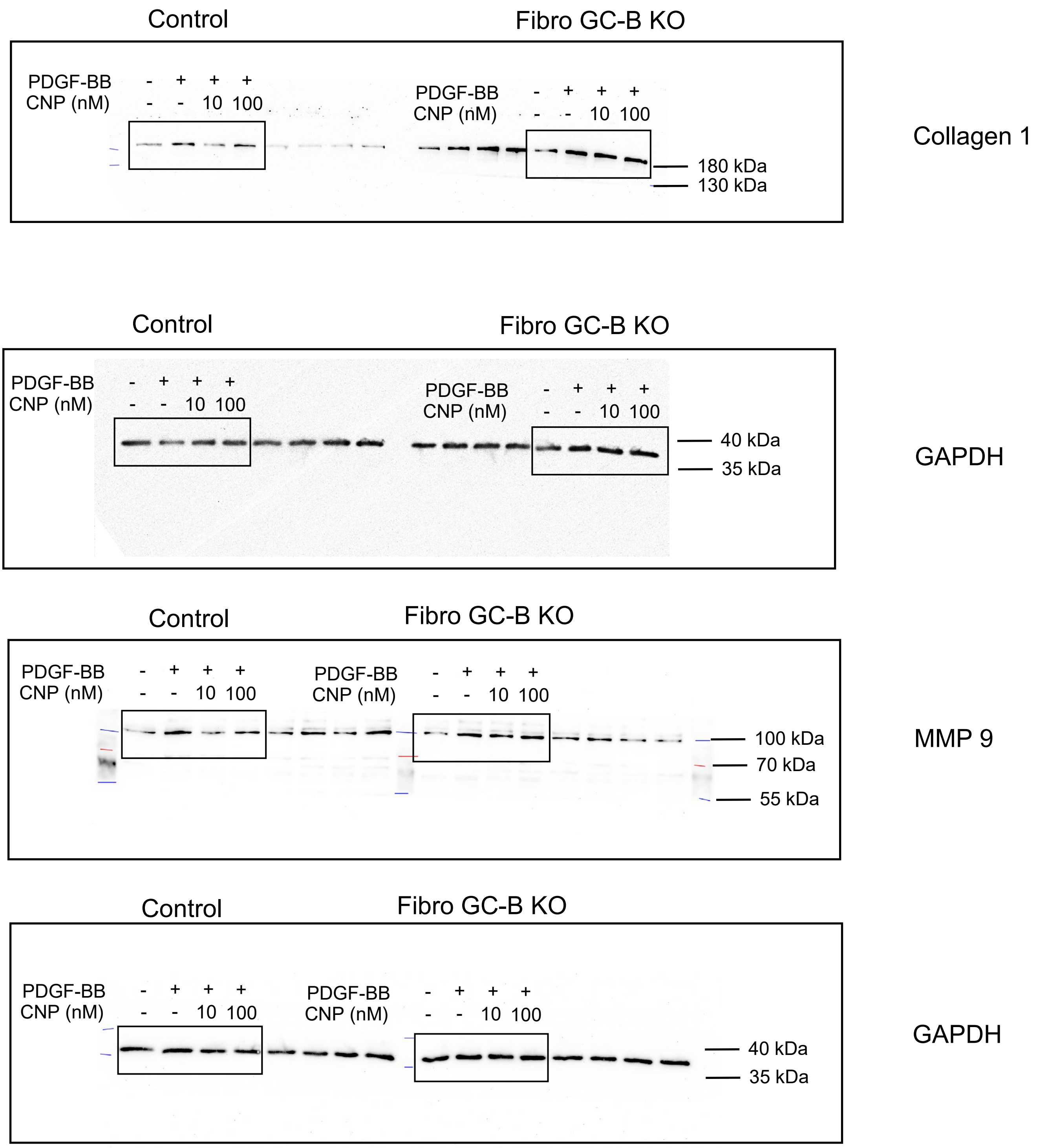

Figure 3B

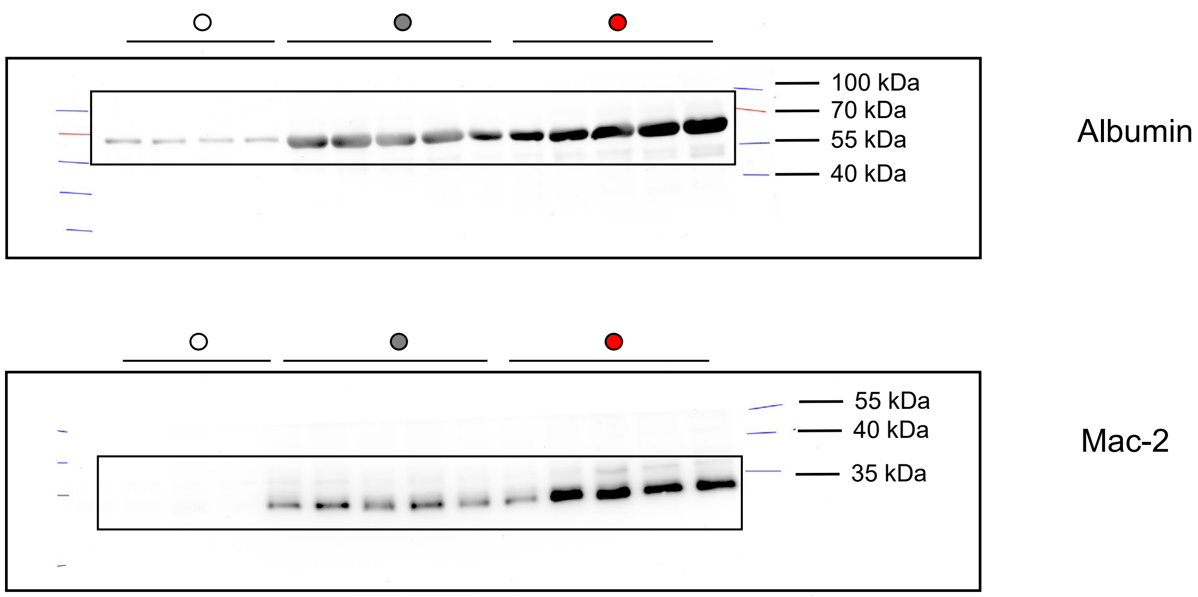

○ Saline-treated control mice
○ Saline-treated Fibro GC-B KO mice
● Bleomycin-treated control mice
● Bleomycin-treated Fibro GC-B KO mice

Figure 4B

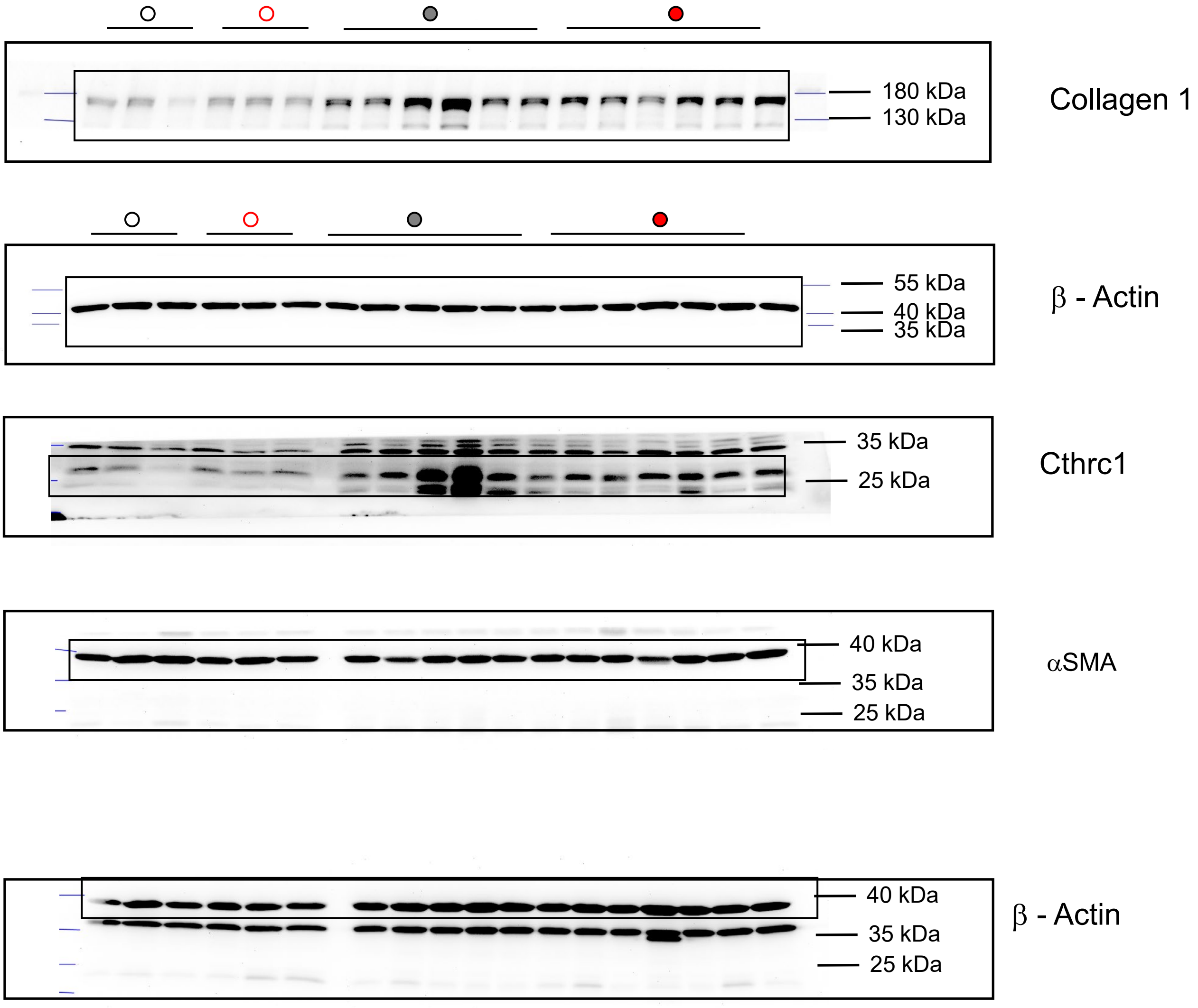

Figure 4B

Saline-treated control mice
Saline-treated Fibro GC-B KO mice
Bleomycin-treated control mice
Bleomycin-treated Fibro GC-B KO mice

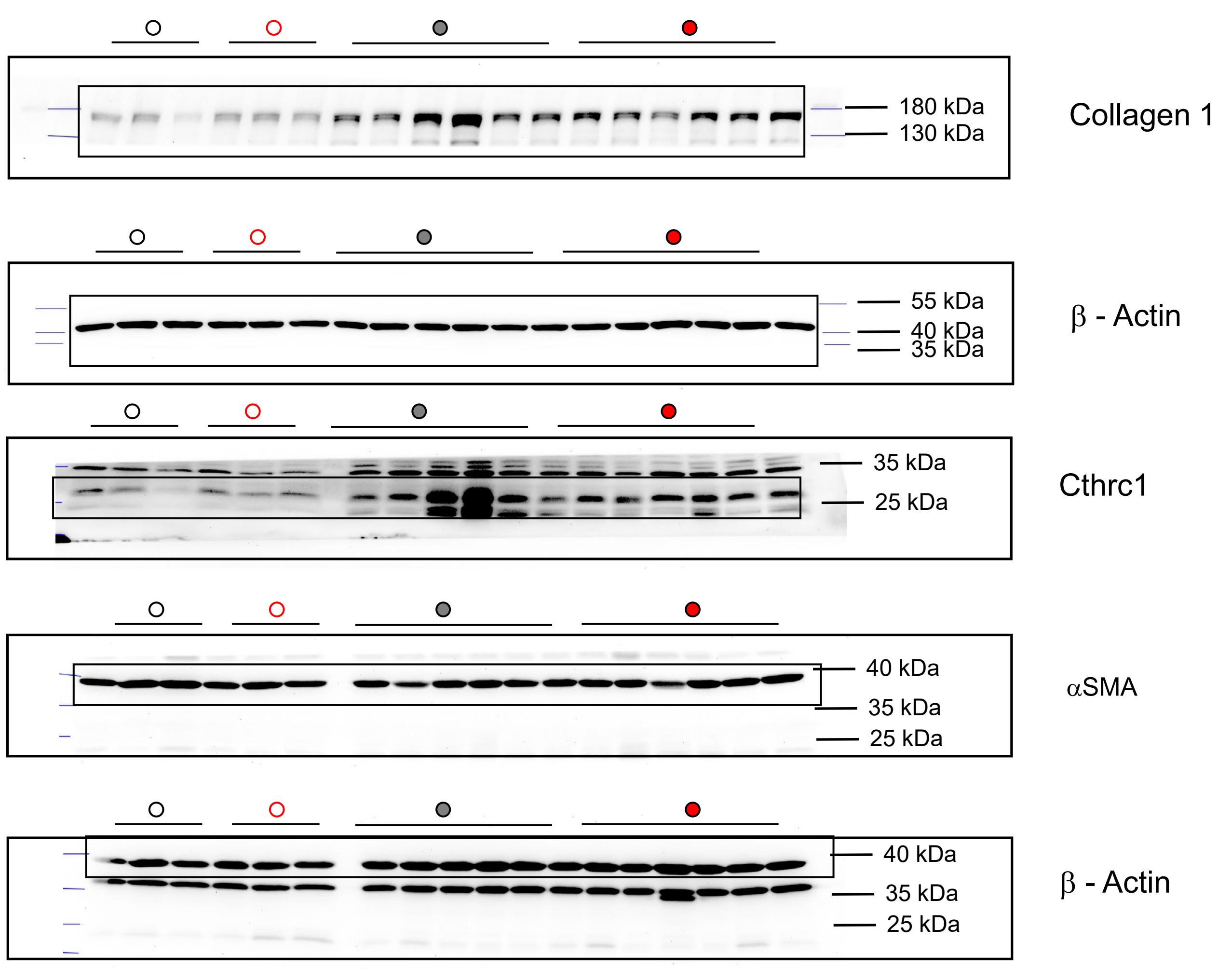

Figure 4C

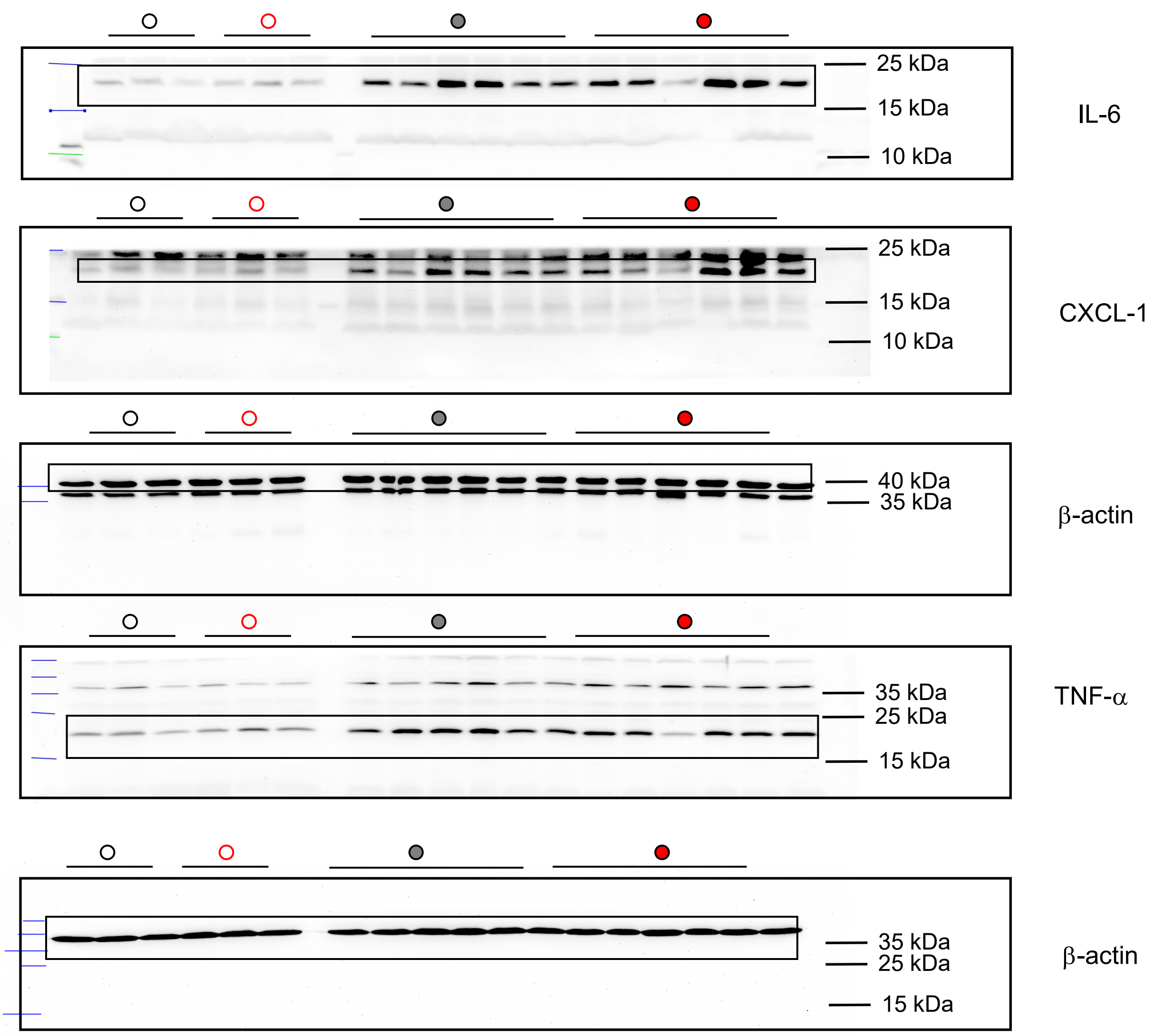

Figure5C

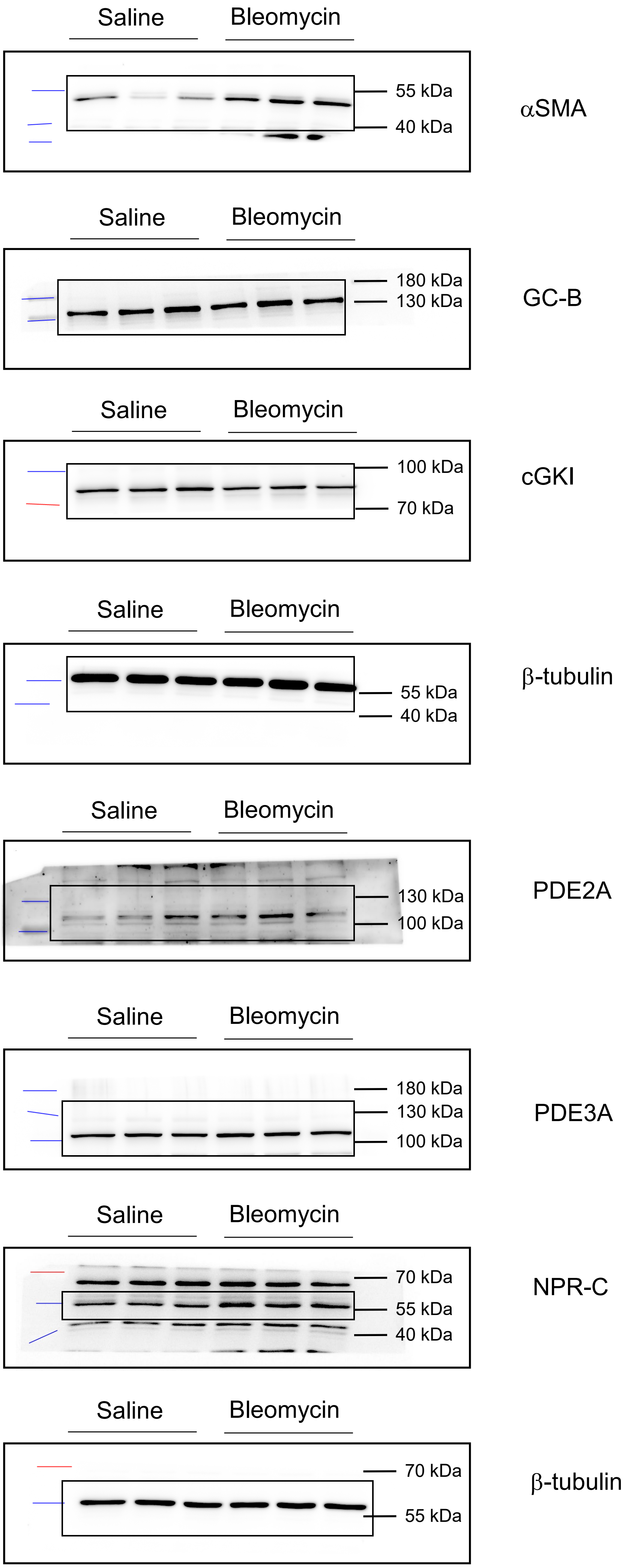
Saline
Bleomycin
55 kDa
40 kDa
αSMA
Saline
Bleomycin
180 kDa
130 kDa
GC-B
Saline
Bleomycin
100 kDa
70 kDa
cGKI
Saline
Bleomycin
55 kDa
40 kDa
β-tubulin
Saline
Bleomycin
130 kDa
100 kDa
PDE2A
Saline
Bleomycin
180 kDa
130 kDa
100 kDa
PDE3A
Saline
Bleomycin
70 kDa
55 kDa
40 kDa
NPR-C
Saline
Bleomycin
70 kDa
55 kDa
β-tubulin

Figure5D

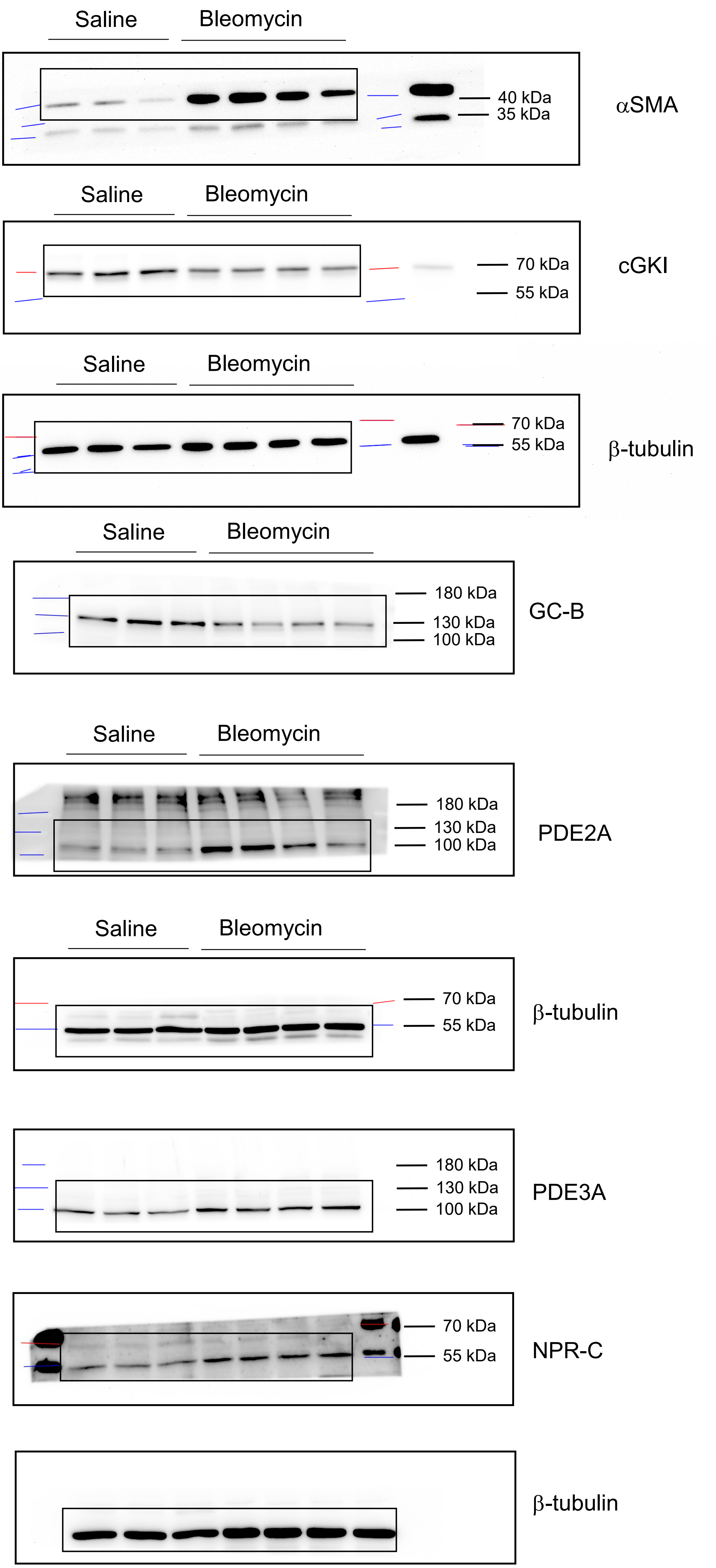
Saline
Bleomycin
40 kDa
35 kDa
αSMA
Saline
Bleomycin
70 kDa
55 kDa
cGKI
Saline
Bleomycin
70 kDa
55 kDa
β-tubulin
Saline
Bleomycin
180 kDa
130 kDa
100 kDa
GC-B
Saline
Bleomycin
180 kDa
130 kDa
100 kDa
PDE2A
Saline
Bleomycin
70 kDa
55 kDa
β-tubulin
180 kDa
130 kDa
100 kDa
PDE3A
70 kDa
55 kDa
NPR-C
β-tubulin

Figure 6A

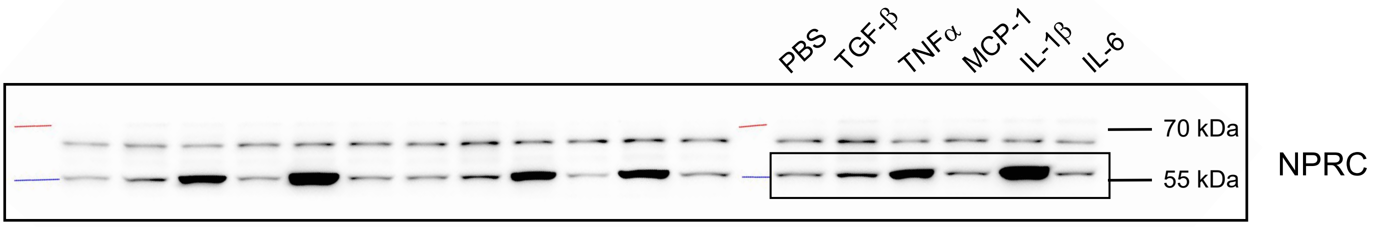

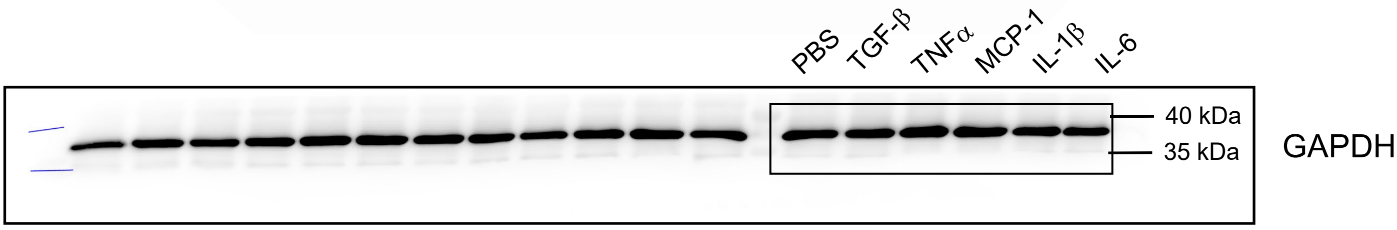

Figure 8A

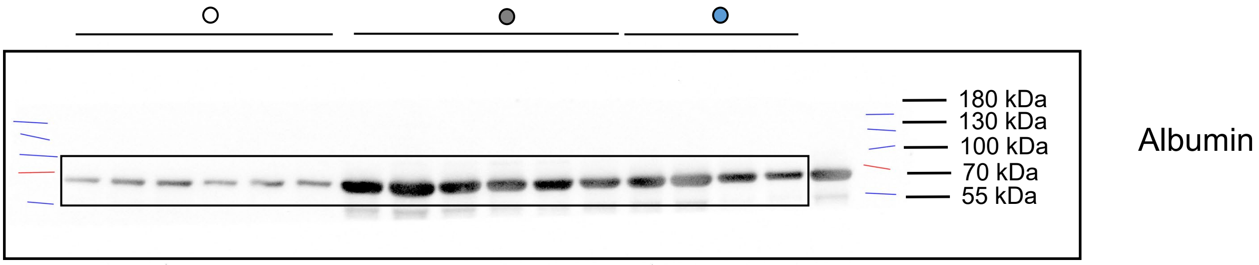

Figure 8B

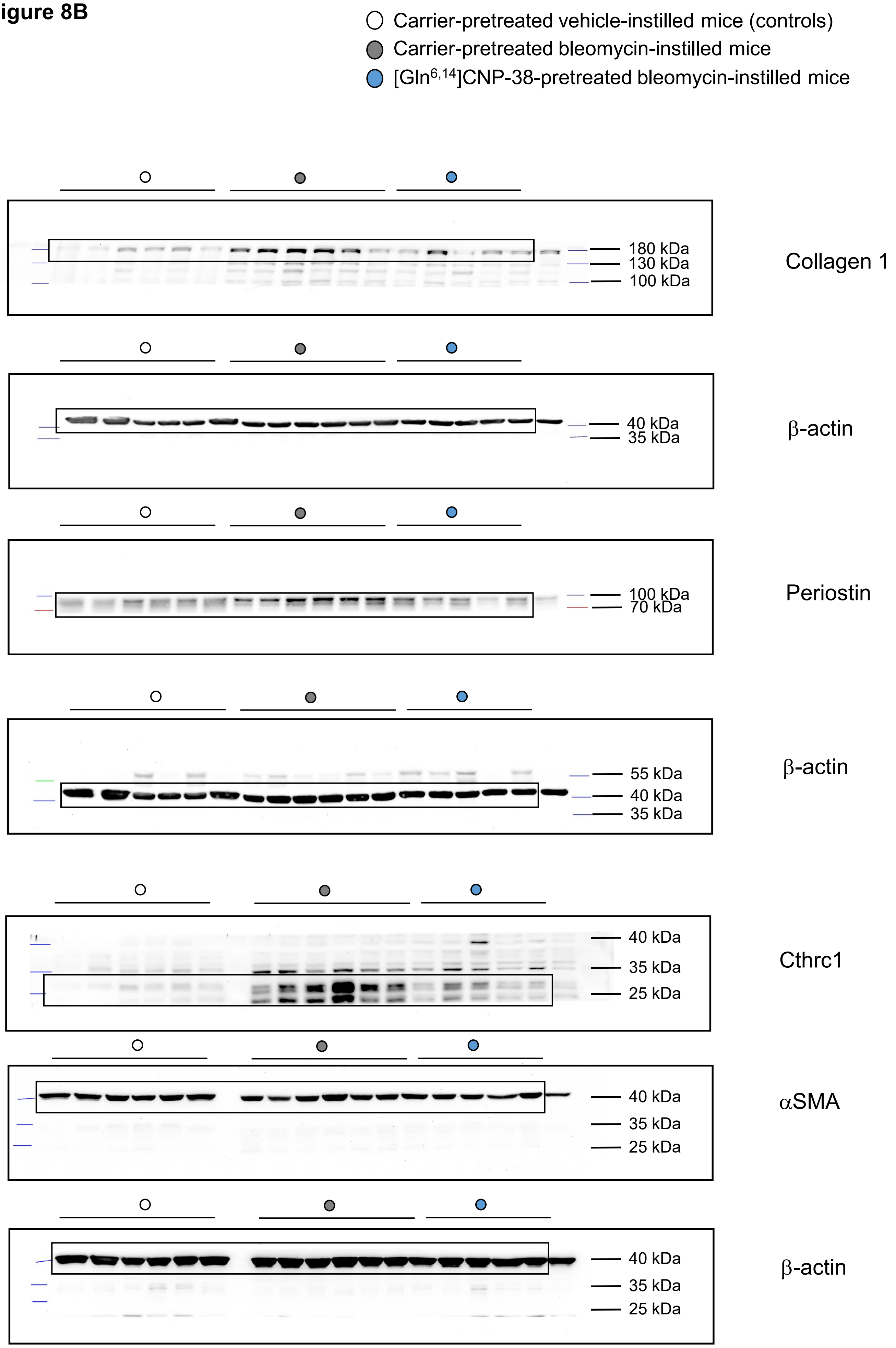
Carrier-pretreated vehicle-instilled mice (controls)
Carrier-pretreated bleomycin-instilled mice
[Gln6,14]CNP-38-pretreated bleomycin-instilled mice
180 kDa
130 kDa
100 kDa
Collagen 1
40 kDa
35 kDa
β-actin
100 kDa
70 kDa
Periostin
55 kDa
40 kDa
35 kDa
β-actin
40 kDa
35 kDa
25 kDa
Cthrc1
40 kDa
35 kDa
25 kDa
αSMA
40 kDa
35 kDa
25 kDa
β-actin

○ Carrier-pretreated vehicle-instilled mice (controls)
● Carrier-pretreated bleomycin-instilled mice
● [Gln[6,14]]CNP-38-pretreated bleomycin-instilled mice

Figure 8B

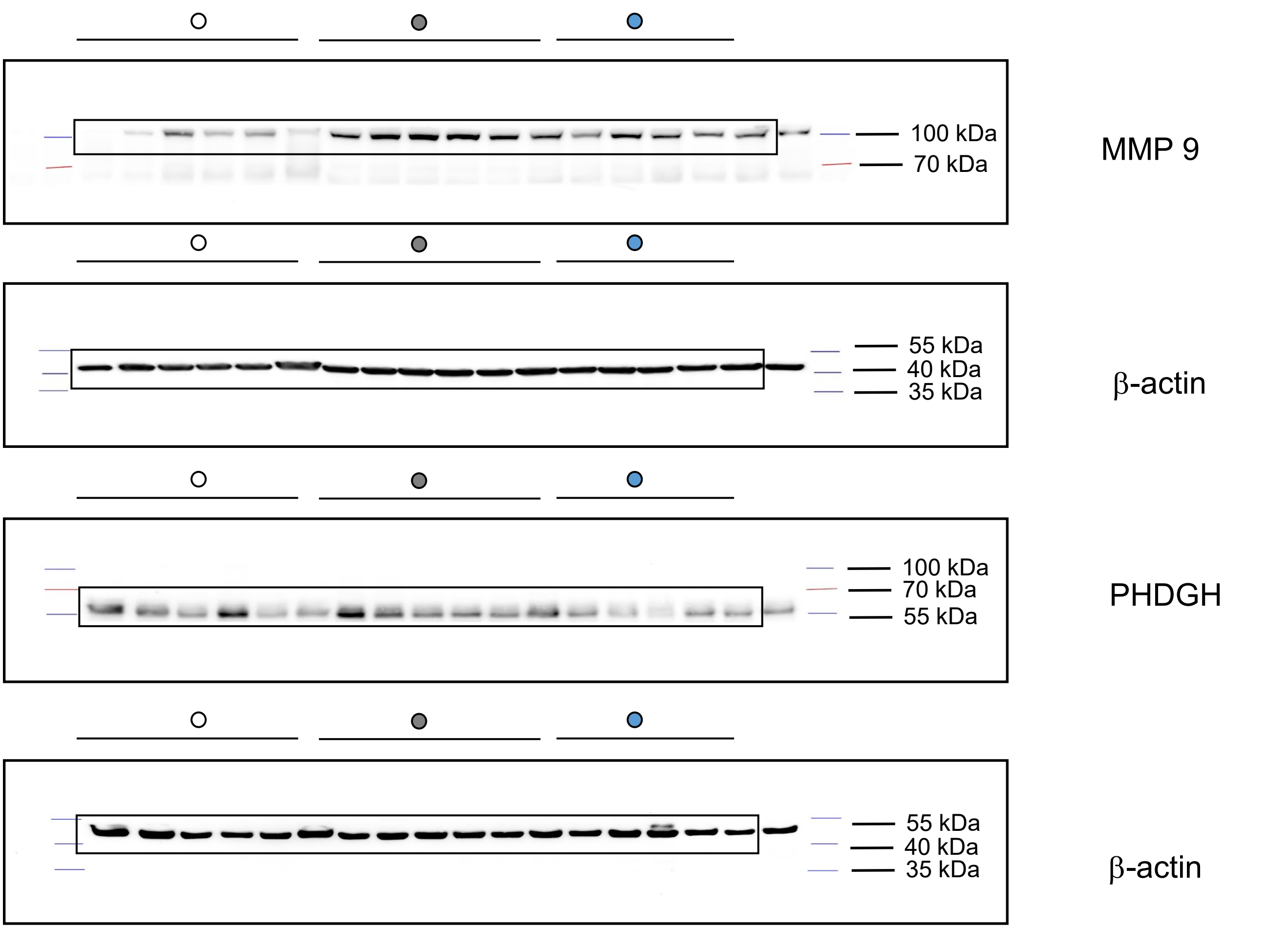

Figure 8C

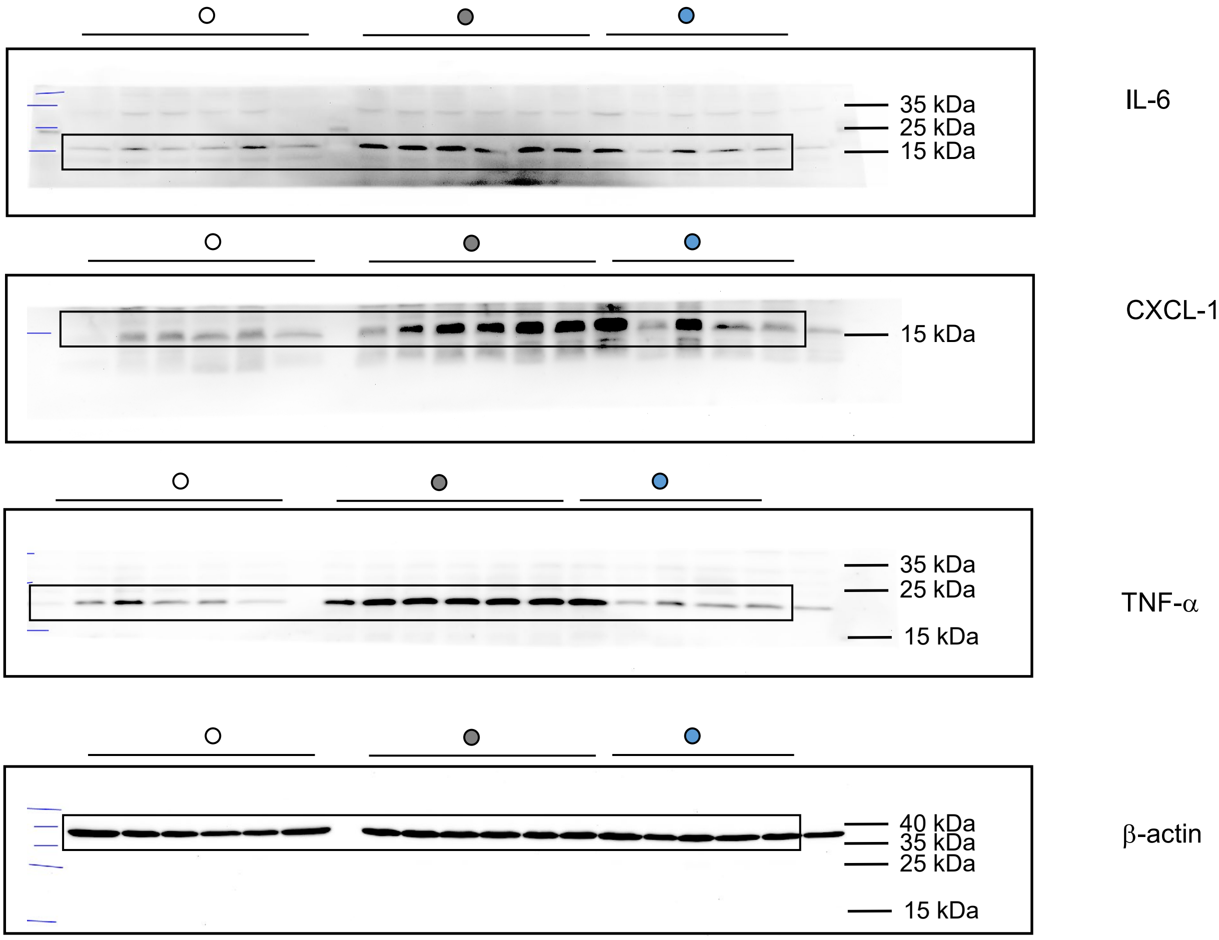

Supplemental Figure 1F

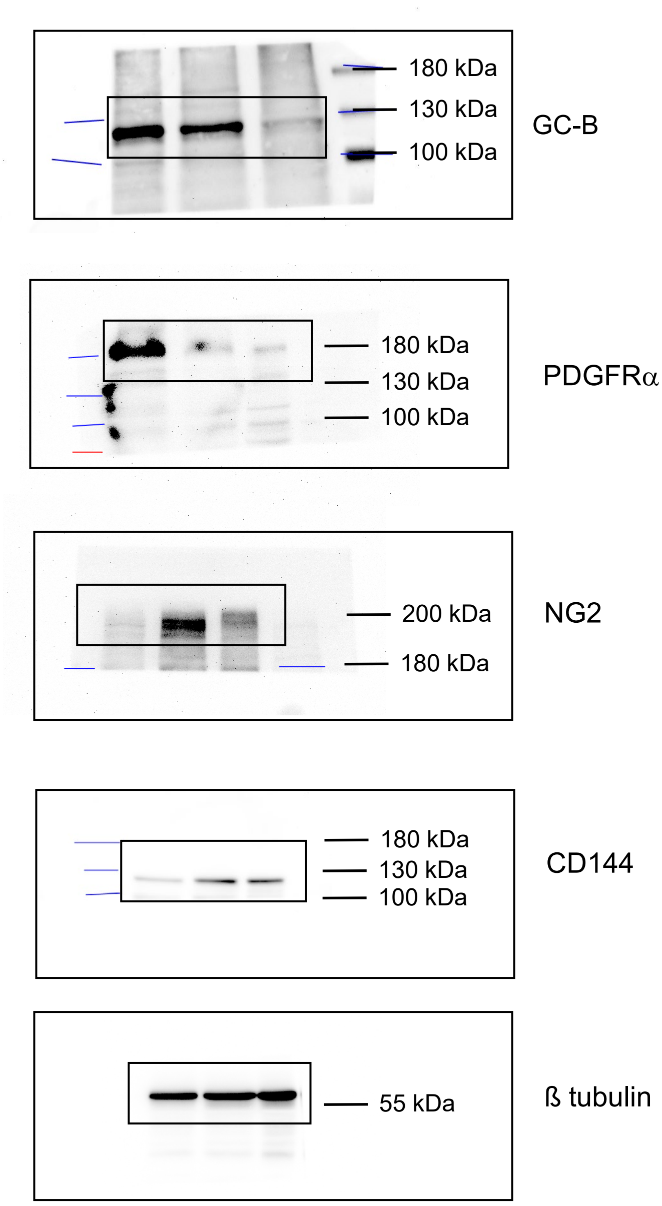

Supplemental Figure 3

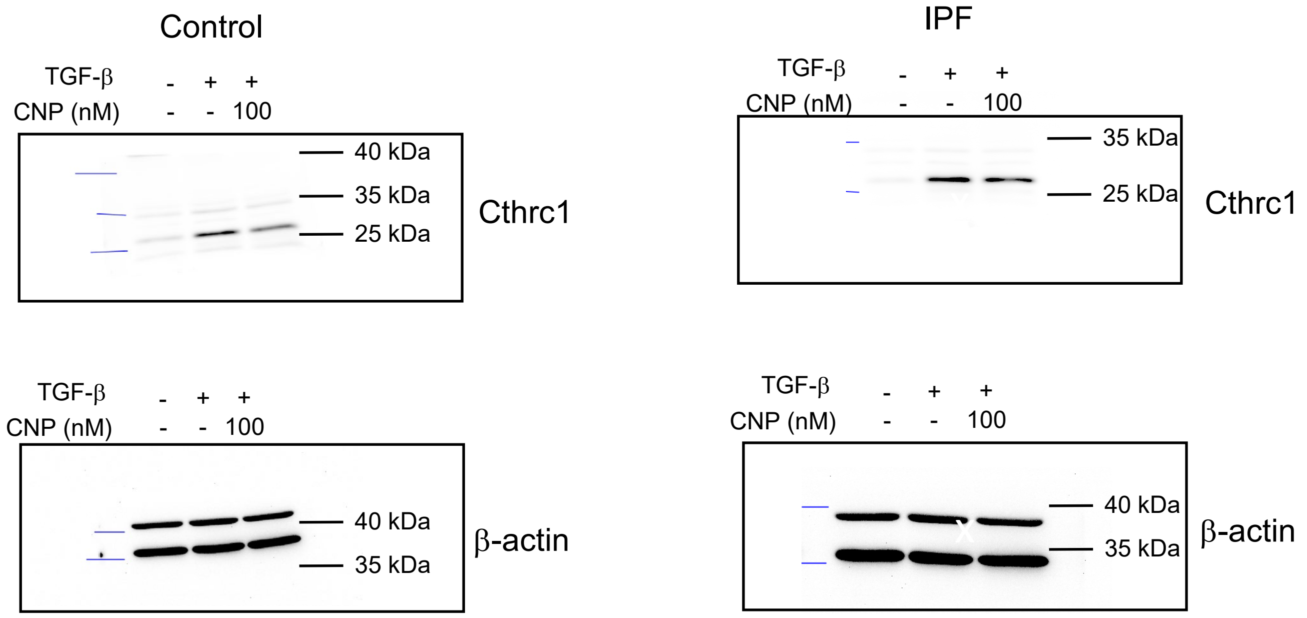

Supplemental Figure 5.

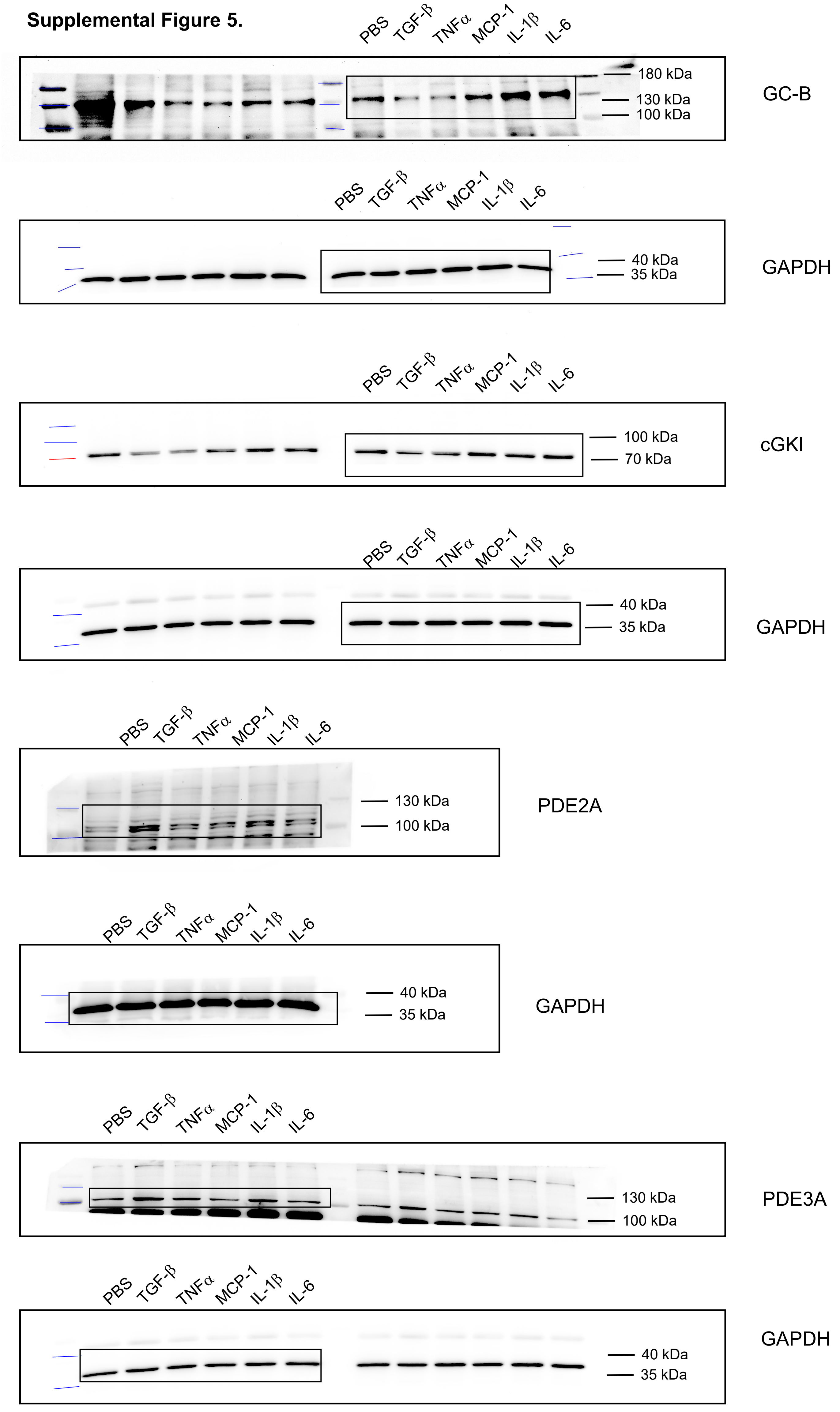

Supplemental Figure 7A

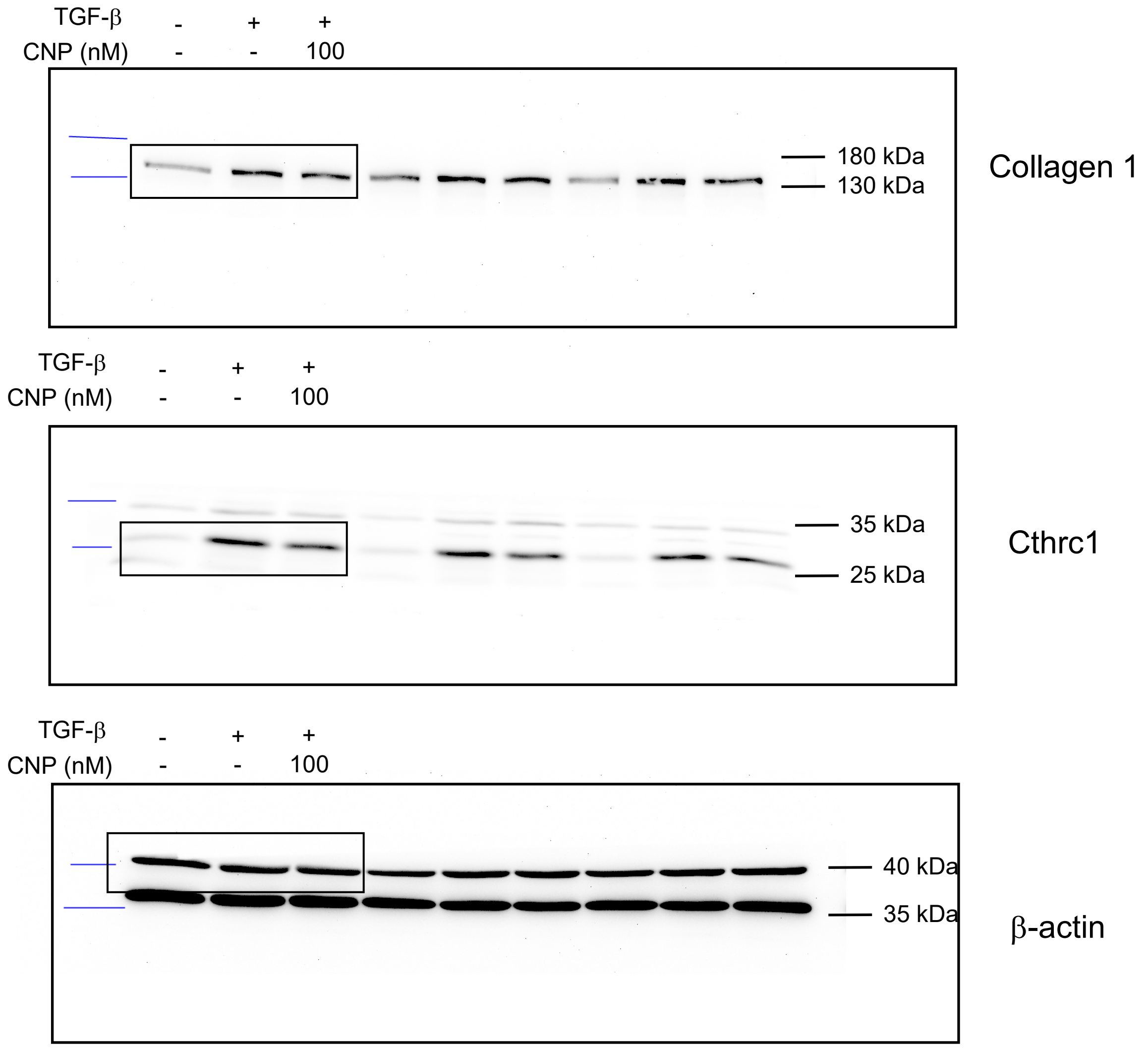

Supplemental Figure 7B

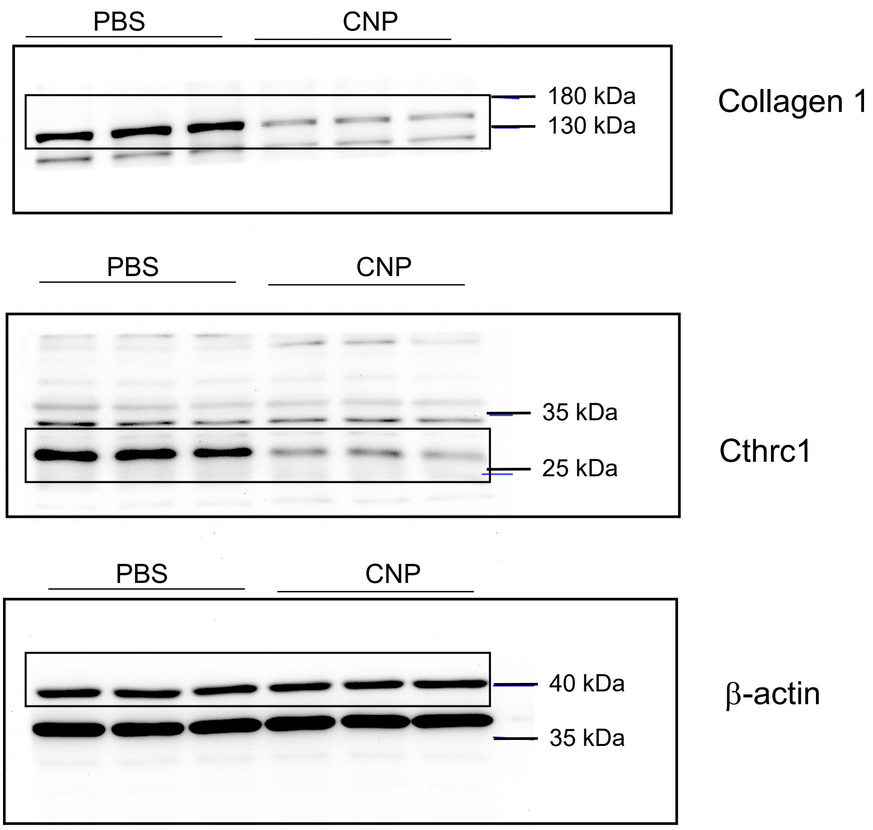

Supplement: Unedited blot and gel images [file jciinsight-11-196812-s138.pdf]
